# Multi-omics characterization of a lytic phage targeting *Listeria monocytogenes*

**DOI:** 10.1128/msystems.00587-25

**Published:** 2025-06-25

**Authors:** Stevan Cucić, Leena Putzeys, Maarten Boon, Dion Lepp, Rob Lavigne, Cezar M. Khursigara, Hany Anany

**Affiliations:** 1 Department of Molecular and Cellular Biology, University of Guelph 3653, Guelph, Ontario, Canada; 2 Agriculture and Agri-Food Canada, Guelph Research and Development Centre 98662 , Guelph, Ontario, Canada; 3 Department of Biosystems, Laboratory of Gene Technology, KU Leuven, Leuven, Belgium; 4 Food Science Department, University of Guelph 3653, Guelph, Ontario, Canada; Kobenhavns Universitet, Frederiksberg C, Denmark

**Keywords:** *Listeria* phage CKA15, multi-omics, phage-host interactions, transcriptomics, proteogenomics

## Abstract

*Listeria monocytogenes* is a foodborne pathogenic bacterium that can persist in food-processing environments. Strictly lytic *Listeria* phages have shown promise as biosanitation and biocontrol agents. However, little is known about the molecular progression of phage expression and the host gene expression profile it elicits in *Listeria*. In this work, the P100-like phage CKA15 was characterized using a proteogenomics-based approach to identify virion-associated proteins, Illumina-based RNA-seq to analyze time-resolved host and phage transcript abundance during infection, and ONT-cappable-seq to experimentally determine the operon structure of the phage genome. We detected 29 phage-encoded putative particle-associated proteins. During infection, a progressive decrease in host transcript abundance and an increase in phage transcript abundance are observed. The progression of phage gene expression indicates a switch in functions from hypothetical at 5 min; nucleic acid metabolism at 15; structural proteins at 25; and DNA packaging, tail assembly, and lysis at 40 min post-infection. Using ONT-cappable-seq, we identified 81 phage transcription start sites (TSS) and 66 transcription termination sites (TTS). We used motif analysis to identify two classes of promoters, corresponding to early and late infection stages. Profound changes in the host transcriptome became evident 5 min post-infection. GO enrichment and KEGG pathway analysis indicate a downregulation of host transcription factor expression and an upregulation of translation, cobalamin biosynthesis, and propanediol metabolism. This research contributes to our systems-level understanding of the infection process of a strictly lytic phage infecting an important foodborne pathogen.

**IMPORTANCE:**

*Listeria monocytogenes* is an important foodborne pathogenic bacterium that contributes to significant mortality worldwide. Since bacteriophages have evolved diverse mechanisms to take over their host bacteria, studying phage interactions with pathogenic bacteria enables researchers to develop novel ways of controlling pathogenic bacteria and tools to study them. Detection of phage particle-associated proteins using mass spectrometry combined with transcriptomic techniques that determine the operon structure of the phage genome, time-resolved transcript abundance of phage, as well as host transcripts, comprises powerful approaches for phage characterization. Moreover, these analyses provide a starting point for hypothesis generation in relation to different aspects of the biology of phages infecting *L. monocytogenes*, including phage particle assembly, gene regulation, host takeover, and bacterial response to phage infection.

## INTRODUCTION

*Listeria monocytogenes* is a pathogenic foodborne bacterial species that causes systemic listeriosis, a severe disease with a high fatality rate. Listeriosis is particularly dangerous for the elderly, immune-compromised people, and young children (1). Because of the severity of the disease and the economic burden of frequent food recalls due to *Listeria* contamination, there is ongoing interest in novel methods to mitigate the threat posed by *L. monocytogenes* in food-processing environments and ready-to-eat foods.

Currently employed methods for biosanitation involve the use of sanitizing agents. However, these can lead to undesirable residues in food as well as upregulating the expression of virulence genes in *L. monocytogenes* (2, 3). An approach for the control of pathogenic foodborne bacteria is the application of bacteriophages (phages) as biosanitizing and biocontrol agents (4). Although phage-mediated biosanitation and biocontrol alone rarely completely eradicate contaminating foodborne pathogenic bacteria, these approaches can be a useful complement to widely used control measures (5). Phages are natural antagonists of bacteria and usually exhibit host specificity at the strain or species level. Most of the lytic non-serotype-specific *Listeria* phages belong to the genus *Pecentumvirus,* which includes phages A511 and P100. Indeed, there are many reports on the efficacy of phages belonging to this genus in reducing *L. monocytogenes* bacterial loads in different food matrices and on surfaces (6–9).

In previous work, we reported that phage CKA15, a member of the genus *Pecentumvirus* closely related to AG20, caused a 2-log reduction of viable counts in *Lm*19111 cultivated under simulated dairy-processing conditions (9). CKA15 and other *Pecentumvirus* members belong to the family *Herelleviridae*. CKA15 has typical morphology for members of its genus, including an icosahedral capsid and a contractile tail (5). This family also includes phage K, a broad-host-range phage infecting *Staphylococcus* spp., and SPO1, which was the object of pioneering studies that led to the discovery of transcriptional cascades (10).

Omics-based characterization of bacteriophages and phage infection contributes to a systems-level understanding of the biosphere’s most abundant biological entity and how phages subvert their host bacterium’s cellular machinery. Recently, transcriptomic studies using novel RNA-seq techniques for phage infection have emerged (11–15). Although previous works have described the proteomic analysis of purified A511 particles (16), as well as extensive reports on *Listeria* lytic phage applications (9, 17–19), endolysins (20), genomics (16, 21–24), and structural studies (25), to our knowledge, there are no published reports of transcriptomic analysis of *L. monocytogenes* infected by a lytic phage.

A powerful starting point for the investigation of phage biology is multi-omics characterization. We set out to determine the proteins encoded in the phage genome that are part of the mature phage particle, the operon structure of the phage genome, the non-coding DNA elements—for example, promoters and terminators—that drive and control the expression of the various genes, the pattern of expression of phage genes at different time points in infection, and the bacterial host physiological state during phage infection. With these data, we derived insights into the potential function of specific gene products, generated hypotheses that can be tested using genetic and biochemical techniques, and identified host processes that may be relevant for phage infection. This work will also enable the discovery of novel synthetic biology parts optimized for the bacterial species under study, as well as the identification of new targets for antimicrobial intervention (26, 27).

## RESULTS

### Growth kinetics of phage CKA15

**Fig 1:**
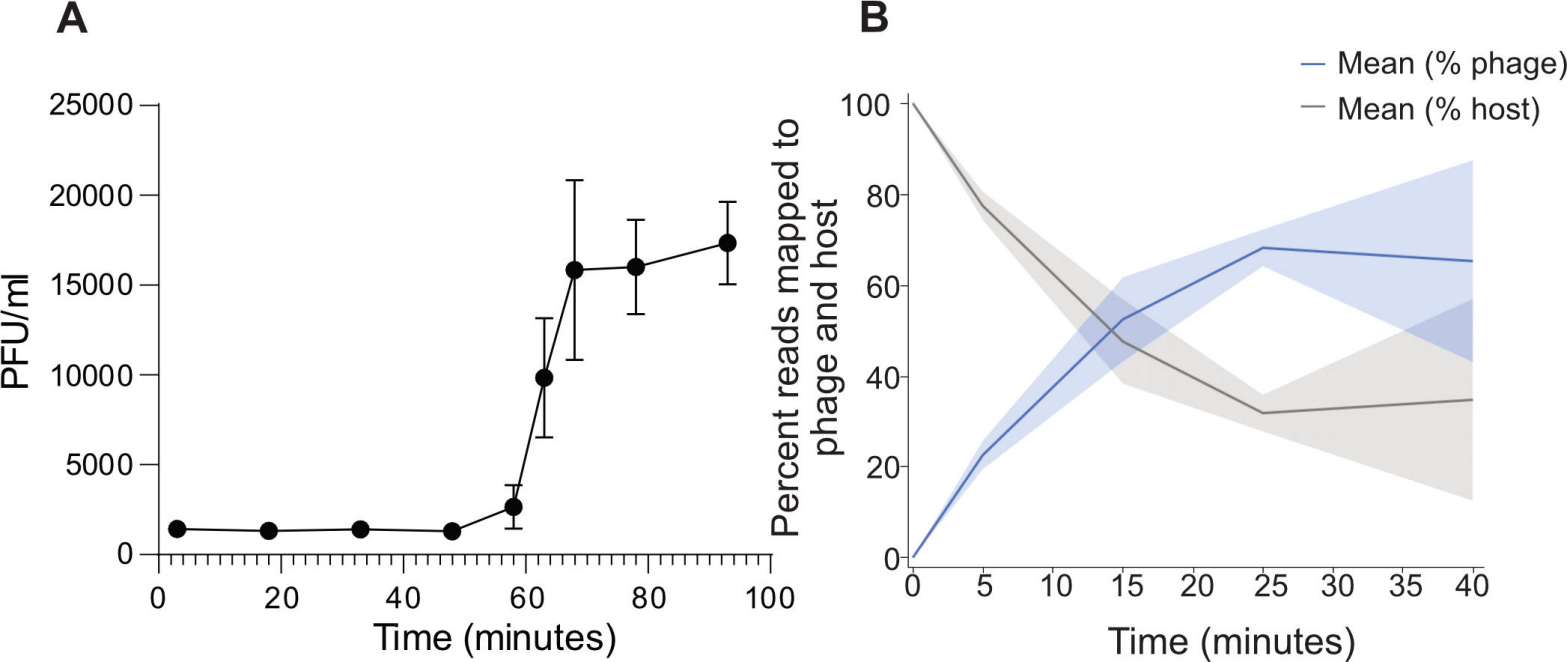
Phage CKA15 one-step growth curve and transcript read mapping during infection. (**A**) *Lm*19111 was grown at 30°C in BHI supplemented with 2 mM CaCl_2_ until an OD600 of 0.8 (*n* = 3). Phage CKA15 was allowed to adsorb for 3 min before diluting the suspension 10^−2^ (tube A) and then a further 10^−1^ dilution (tube B). On this graph, T = 0 is set as the moment of adding phage to the adsorption tube. The error bars represent the standard deviation of the mean from three biological replicates. The phage titer was standardized to tube A. More details are in Materials and Methods and in Table S1. (**B**) Percentage of transcripts mapped to phage and host genomes at different infection time points. Shaded area indicates the standard deviation of the mean (*n* = 3).

Phage CKA15 is a strictly lytic phage belonging to the genus *Pecentumvirus*, a myovirus with typical morphology for members of the genus, including an icosahedral head and a contractile tail (16). Its linear dsDNA genome is 137,077 bp in length and contains long terminal repeats that are 3,134 bp long (9). Our group has previously reported on the phenotypic characteristics of this phage, particularly those pertaining to biofilm degradation (9). Under optimal growth conditions, this phage has a latent period of 50 min and a burst size of approximately 14 (Fig. 1A; Table S1). The frequency of CKA15-resistant *Lm*19111 mutants is 4.5 × 10^−5^ ± 3×10^−5^ (Table S2), which is lower than some other phage-bacteria systems (28, 29). Phages in this genus bind to wall teichoic acids on the cell envelopes of permissive hosts (21). The abundance of this cell wall polysaccharide and the affinity of adsorption-associated *Pecentumvirus* tail proteins to WTA enable the relatively synchronous infection at high cell densities, making it suitable for transcriptome characterization.

### Proteogenomic identification of phage CKA15 structural proteins and other potential virion-associated proteins

In the first step, we performed mass spectrometry on purified phage particles to identify phage proteins incorporated into the mature particles. Phage CKA15 particles were purified by two rounds of CsCl gradient centrifugation and concentrated to a concentration of 2 × 10^12^ PFU/mL before separating the phage polypeptides using SDS-PAGE, performing in-gel trypsinization, and analyzing tryptic peptides by ESI-MS/MS (Fig. S1A). The detected peptides were then matched to a database of all possible polypeptides greater than 30 amino acids long encoded in all six frames of the phage genome (30). We detected 29 phage proteins, most encoded in the predicted structure and assembly module of the phage genome (Table 1). Nine detected proteins are encoded by genes previously annotated as encoding structural proteins. These include a tail tape measure protein with predicted lytic activity, a tail-associated lysin, a portal protein, a tail sheath protein, two baseplate-associated proteins, the major capsid protein, a receptor binding protein, and a tail tube protein. The ten remaining proteins are hypothetical proteins in the structure and assembly module. We also detected the predicted pro-head protease, which may have been present in residual quantities detectable by mass spectrometry in the phage capsids. Furthermore, nine detected proteins are not in the structure and assembly module. These included the phage endolysin and a protein—encoded by a CDS with locus tag CKA15_055—predicted by HHPred (31) to have RNA binding activity. This gene is located upstream of a gene predicted to encode a transcriptional regulator and downstream of the part of the phage genome encoding tRNAs. Furthermore, a coding sequence located on the long terminal repeat encodes one of the proteins detected in mature particles (locus tag CKA15_006).

**TABLE 1:** Phage proteins in purified mature phage particles detected by ESI-MS/MS^*a*^

Protein IDs	Molecular weight (kDa)	Peptide count	Sequence coverage (%)
CKA15_123	146.05	24	25.5
TMP/tail lysin 1	131.03	27	25.9
CKA15_125	128.35	62	78.7
Tail lysin 2	90.306	15	35.2
Portal protein	61.592	26	53.9
Tail sheath protein	61.412	37	79.8
Baseplate component	57.605	3	10.2
CKA15_055 *	56.784	20	57.1
Major capsid protein	51.482	41	79.7
Receptor binding protein	46.69	12	41.7
Baseplate protein	40.05	11	40
Endolysin *	37.71	8	31.1
CKA15_107	33.369	12	53.2
CKA15_110	32.285	3	9.1
CKA15_094 *	32.013	10	45.2
CKA15_108	31.228	12	38.2
Prohead protease	29.65	4	29.5
CKA15_119	27.419	16	75.6
CKA15_121	26.421	9	51.4
CKA15_162 *	26.337	7	40.7
CKA15_120	21.45	3	17
CKA15_124	19.381	6	38.9
CKA15_183 *	19.092	10	86.7
Tail tube protein	18.715	3	17.1
CKA15_140 *	16.87	2	25.3
CKA15_128	16.681	11	69.8
CKA15_188 *	11.896	2	28
CKA15_006 *	11.009	2	33.3
CKA15_084 *	10.355	6	70.7

^*a*^ Proteins indicated by an asterisk are encoded by genes that are not in the structure and assembly module of phage CKA15.

**Fig 2:**
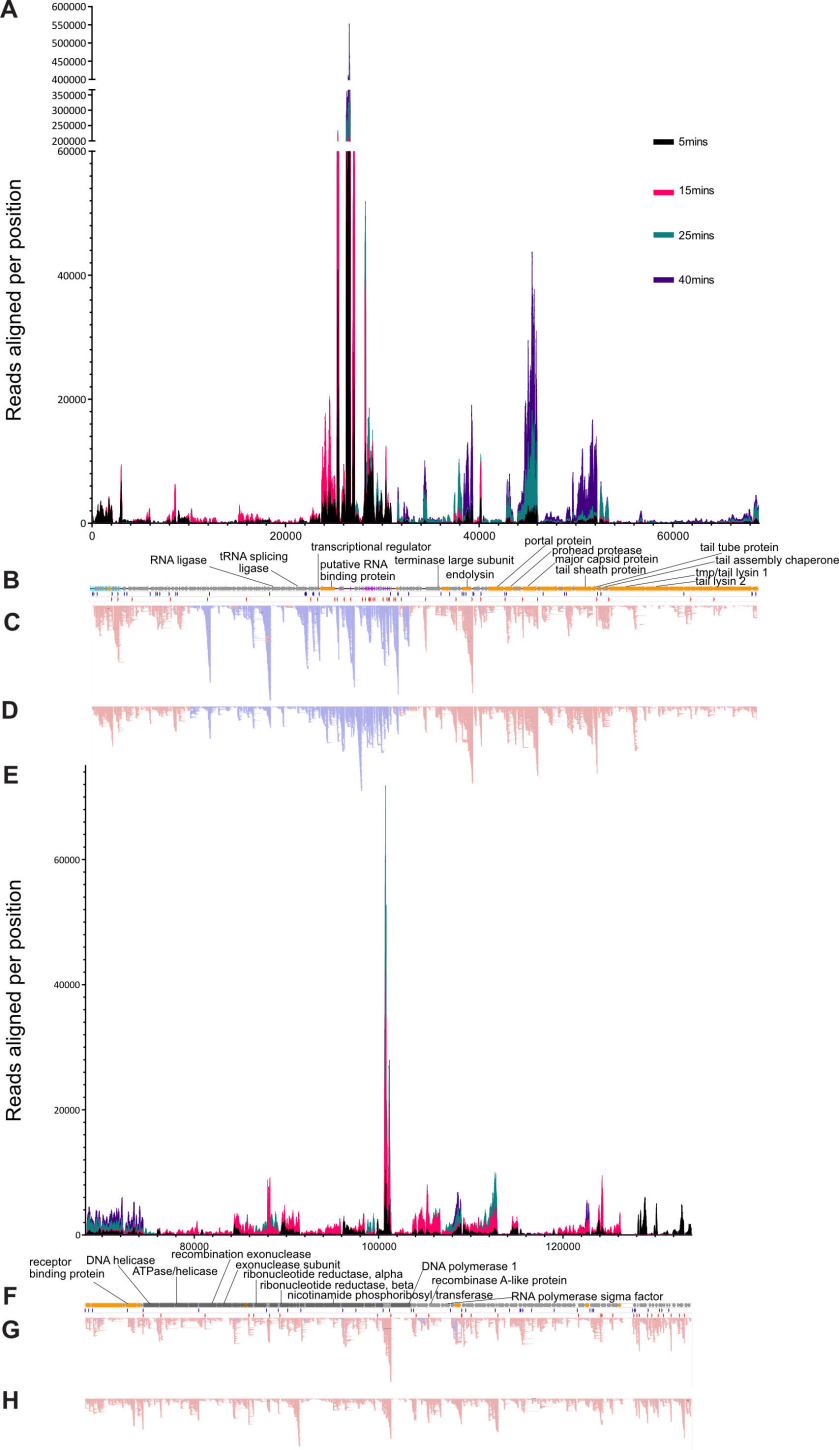
Phage CKA15 multi-omics. (A and E) Sequencing reads from time-resolved RNA-seq experiments aligned per nucleotide position in phage genome (*n* = 3). (**B and F**) Linear genome plot with coding sequences encoding proteins detected in purified particles colored in orange. LTR genes are highlighted in teal. The structure and assembly module is highlighted in light orange. DNA replication module is highlighted in gray. Blue and red arrows indicate transcriptional start and stop sites, respectively. (**C and G**) Primary transcript enriched reads from ONT-cappable-seq experiment. (**D and H**) Reads from ONT-cappable-seq experiment without primary transcript enrichment. (C, D, G, and H) Reads colored in pink aligned to the Watson strand. The reads are colored in blue and aligned to the crick strand. (**A, B, C, and D**) Position 1–68,900 in the CKA15 genome. (E, F, G, and H) Position 68,200–133,943 in the CKA15 genome.

HHPred analysis suggests that CKA15_055 encodes an RNA-binding protein. This protein is encoded in a region of the genome between 620 bp and 5.64 kbp downstream of the region that contains the phage’s tRNA genes and is transcribed from the same strand (Fig. 2B).

Previously, researchers have identified phage structural proteins with enzymatic activity (32–34). In tailed phages, there are peptidoglycan (PG)-degrading enzymes that enable DNA translocation into host cells—virion-associated lysins—and those that liberate progeny phage following assembly and DNA packaging—the endolysins. Bioinformatic analysis using InterProScan (35) suggests that the genome of phage CKA15 encodes PG-degrading enzymes with three distinct types of activity, including predicted amidase, glycosidase, and endopeptidase. All three proteins were detected in the mass spectrometry data from CsCl-purified phage particles. However, zymography experiments (36) revealed PG-degrading activity in the mature phage particles for only two clearing zones, both of which correspond to the CKA15 endolysin and a degradation byproduct of the endolysin consisting solely of the enzymatic domain (Fig. S1B).

### Transcriptomic profiling of CKA15-host interaction reveals phage infection stages

We performed RNA-seq analysis on uninfected and infected cells to assess the progression of phage infection and the physiological state of the host *L. monocytogenes* ATCC strain 19111 (*Lm*19111) during infection of logarithmic phase *Lm*19111 by *Pecentumvirus* CKA15. Cell densities of 5 × 10^8^ CFU/mL and phage titers of 3 × 10^9^ PFU/mL were used, which, given the rapid adsorption kinetics of CKA15, ensures a relatively synchronous infection. The actual MOI at 3 min was empirically validated by measuring the proportion of bacterial survivors. Three minutes after the addition of phage, 98% of the bacteria were infected by phage, which is consistent with an MOI of 4 (Table S3) (37).

To assess the quality of the RNA-seq data, we performed principal component analysis (PCA) and read mapping to host and phage genomes. As expected, the PCA analysis showed clustering of replicates by time point, although the data from the 15-min time point were more variable than the other post-infection time points (Fig. S2). The percentage of reads mapped to the host and phage genomes during infection also matched expectations, with the ratio of phage:host reads increasing throughout the infection. This percentage of reads mapped to the phage genome reached a maximum of ~70% by 25 min post-infection (Fig. 1B).

### Phage CKA15 exhibits heterogeneous transcriptional activity across its genome

There was a high level of heterogeneity in phage transcriptional activity across the phage genome (Fig. 2A). CKA15_059 had the most reads aligned to the phage genome of any annotated phage coding sequence (CDS) at all times post-infection. CKA15_059 and CKA15_060 had the most transcriptional activity of any of the phage genes in CKA15, by far, with transcripts per million (TPM) normalized transcript abundance accounting for between 7% and 34% of all transcripts aligned to either phage or host genes and with the proportion of reads increasing as infection progressed. By 40 min post-infection, approximately 67% of the phage transcripts were aligned to these two genes. However, given that CKA15_059-061 are small open-reading frames (ORFs) that lack obvious ribosome binding sites, and given that we found no homologs of the putative proteins in GenBank using BlastP, it is likely that these three ORFs are not CDSs. We previously annotated these as protein-coding regions under the assumption of a high density of coding sequences in phage genomes. However, the absence of prominent ribosome-binding sites and the presence of promoter sequences is consistent with these loci encoding non-coding RNAs.

Another feature of the transcriptional activity mapped to the genome of phage CKA15 is a relative transcriptional dead zone, which comprises 1.2 k bp and is located immediately downstream of CKA15_188. The latter encodes a 96 amino acid basic protein (38) of unknown function (predicted to have a charge of 3 at neutral pH) that we detected in purified particles. The 1.2 kbp region downstream of CKA15_188 also lacks discernible coding sequences. Beginning with the TGA stop codon of CKA15_188, there is a repetitive sequence composed of (TAGGA)_27_, followed by a (TAGGA)_33_ repeat, with the two repetitive regions separated by 30 bp. Furthermore, 785 bp downstream of the second repetitive region is a 185 bp segment of the genome that has an AT content of 80%, compared with 65% for the genome as a whole, suggesting that this may be an origin of replication of the phage gDNA, similar to what has been reported for phage K (39).

### Different functional categories of phage genes are associated with different stages of infection

**Fig 3:**
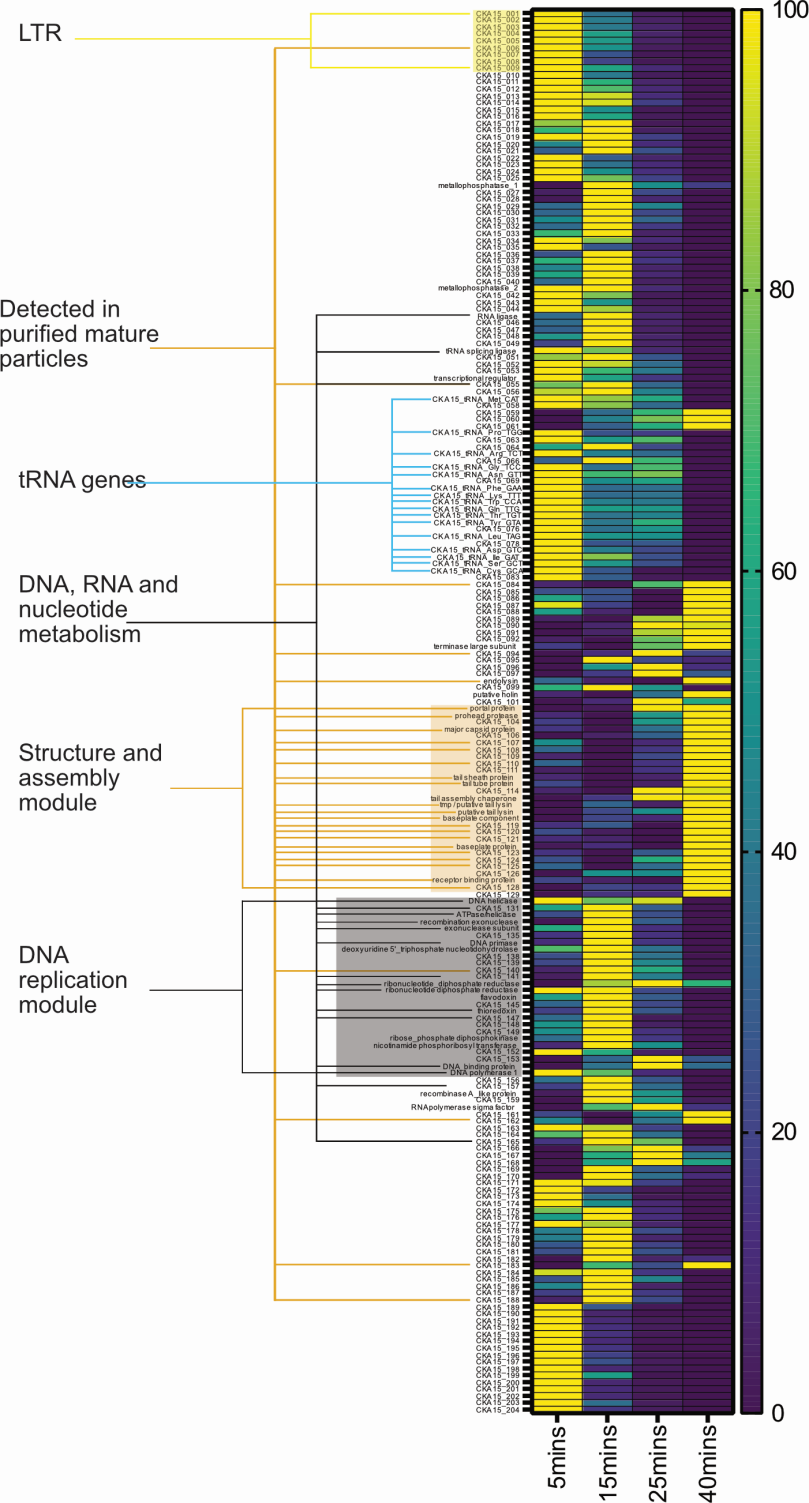
Global transcriptional heatmap of phage transcript abundance at different time points post-infection. Global transcriptional heatmap showing min-max normalized transcript per million values for phage genes. Long terminal repeat, structure and assembly module, and DNA replication module are highlighted in yellow, orange, and gray, respectively. Orange lines indicate genes encoding proteins detected in mature phage particles.

Although most of the proteins encoded by CKA15 are annotated as hypothetical, the timing of maximal expression of the corresponding genes provides clues toward their function because the phage replicative cycle follows a coherent sequence of events, which proceeds from host takeover, phage gDNA replication, assembly of the mature phage particle, and finally lysis of the host cell. Most genes encoding proteins detected in purified particles had maximum expression levels at 40 min and were in the structure/assembly module (Fig. 2A and E). Conversely, genes encoded in the DNA replication module and gene-assigned functions related to DNA, RNA, and nucleotide metabolism had maximal expression at 15 min. The genes on the long terminal repeats had maximum mean expression at 5 min. The only exception was CKA15_009, which had a slightly higher mean expression at 15 min compared with 5 min (Fig. 3). However, based on our differential analysis of the expression of phage genes, none of the genes found on the long terminal repeats had significantly different expression levels at 5 compared with 15 min. Nevertheless, the elevated level of gene expression of the long terminal repeat (LTR) genes at 5 min compared with later infection is consistent with their functions related to host take-over. This matches expectations based on previous work identifying early genes in the canonical phage SPO1 (40). Like *Pecentumvirus* members, SPO1 belongs to the Family *Herelleviridae* and has a similar genome organization to that of P100-like phages (16).

### Phage genes with maximum expression at 5 min lack biochemically characterized orthologs

**Fig 4:**
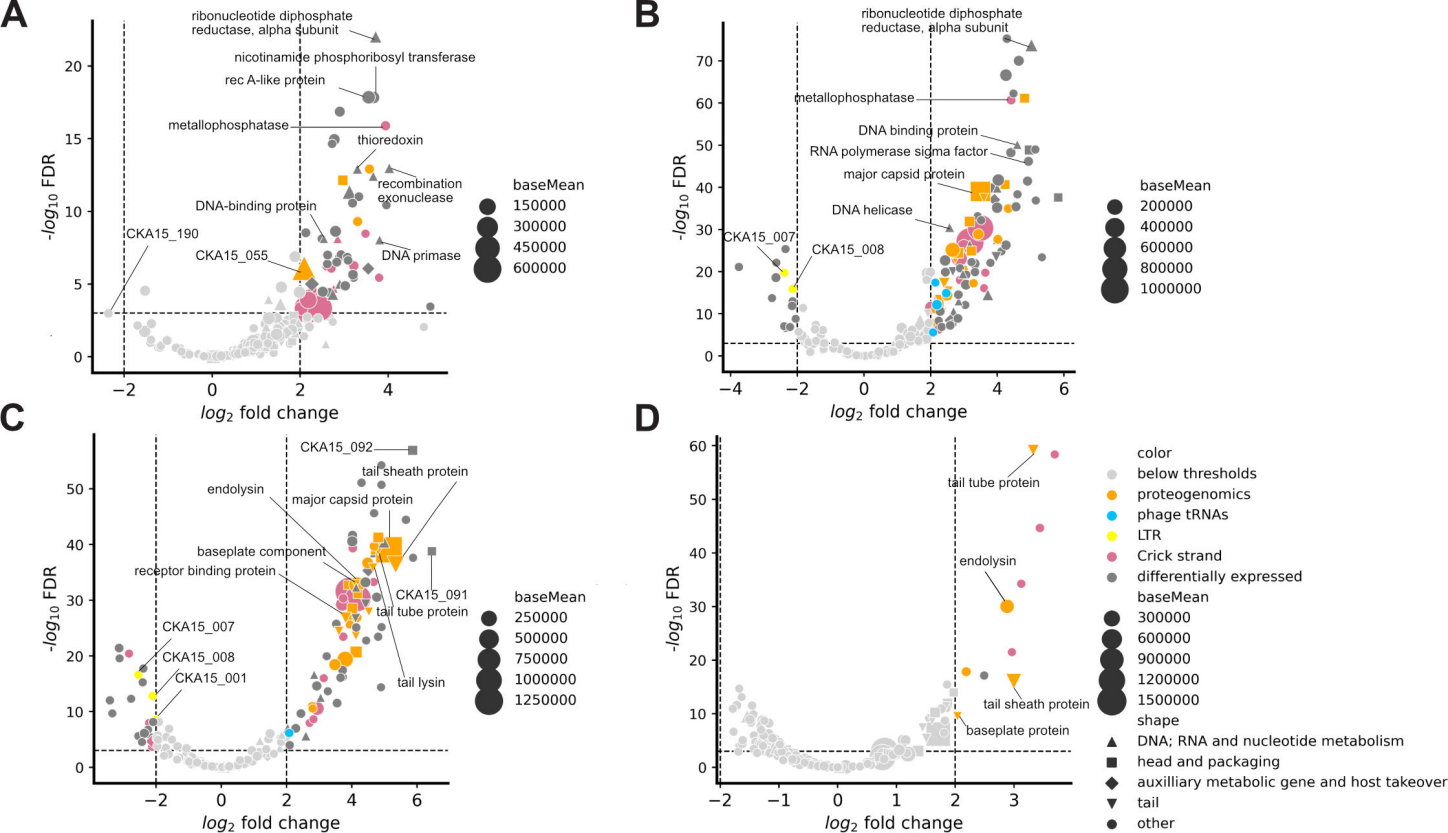
Pairwise comparisons of phage transcript abundance at different time points post-infection. (**A–D**) Volcano plots showing pairwise comparisons of phage transcript levels. (**A**) Five vs 15 mins; (**B**) 5 vs 25 mins; (**C**) 5 vs 40 min; and (**D**) 25 vs 40 min.

There was a cluster of genes around CKA15_190 that had maximum mean TPM values at 5 min. Indeed, CKA15_190 is the only gene that passed the log2 fold change cutoff of −2 for consideration as significantly more expressed at 5 min compared with 15 min (Fig. 4A). CKA15_190 is also enriched approximately 4-fold at 5 versus 25 min. This gene is predicted to encode a small protein, only 49 amino acids, that is highly acidic (predicted charge between −10 and −11 at neutral pH) (36). Three other genes—CKA15_200, CKA15_191, and CKA15_202—had low adjusted *P* values (0.0072, 0.0185, and 2.89 × 10^−5^, respectively) in this pairwise comparison and had log_2_ fold change values of, respectively, −1.7, −1.53, and −1.52. Additional genes with maximal expression at 5 min post-infection include CKA15_173 and CKA15_174 (Fig. 3). These two genes are part of the same transcriptional unit (details below). SignalP (41) predicts CKA15_173 to encode a protein with a lipoprotein signal peptide sequence. CKA15_174, immediately downstream of 173, is abundant early in infection, with an average TPM value at 5 min of 1,440 ± 683.

### Phage genes with maximum expression in the middle stages of infection include many with predicted functions associated with DNA and RNA metabolism

In the pairwise comparison between 5 and 15 min post-infection, we see that most of the genes significantly upregulated at 15 min are part of the DNA replication module (Fig. 4A). These include genes encoding recombinase A-like protein, DNA-binding protein, recombination exonuclease, DNA primase, ribonucleotide reductase, nicotinamide phosphoribosyl transferase (NMPT), and ribose phosphate diphosphokinase. The products of the latter two genes may catalyze sequential steps in the pentose phosphate pathway and thereby play roles in purine and pyrimidine metabolism as well as NAD scavenging (42).

The differential expression analysis comparing the 25- and 5-min time points reveals upregulation of phage tRNA genes relative to 5 min post-infection (Fig. 4B). The genes upregulated at 25 min versus 5 also include those with predicted functions related to DNA, RNA, and nucleotide metabolism, head and packaging, tail, and auxiliary metabolic genes. Comparing these time points, we see the expression of genes encoding proteins detected in our proteogenomics experiment, including major capsid and tail-associated proteins (Fig. 4B).

### Phage genes with maximum expression in late infection encode predicted structural, assembly, and lysis-related proteins

With the 5-min versus 40-min pairwise comparison (Fig. 4C), we observe that a gene (CKA15_091) that Pharokka annotated as a terminase large subunit is one of the most enriched genes late in infection. However, there is a larger downstream gene Pharokka annotated the same way as well as an upstream gene that is larger than CKA15_092, which was annotated as terminase small subunit. To avoid confusion, we opted to annotate CKA15_091 and 092 as hypothetical. However, CKA15_091, 092, and 093, the latter of which we annotated as encoding terminase large subunit, are among the most upregulated genes at 40 min compared with 5 min post-infection. However, these three proteins were not detected in the purified particles, consistent with a role in particle assembly or DNA packaging but not with structural proteins.

In the 25- versus 40-min pairwise comparison (Fig. 4D), highly upregulated genes at 40 min include CKA15_085, 86, 87, and 88. These genes are transcribed in the reverse direction and are not in the structure and assembly module. However, their TPM values at 40 min are lower, between 10-fold and 100-fold, than genes encoding tail tube, tail sheath, and endolysin (CKA15_113, 112, and 98, respectively). Notably, the differential expression of the gene encoding major capsid protein does not reach the log_2_ fold change (L2FC) threshold in this pairwise comparison. Still, three of the tail-associated genes do, including those encoding the tail tube and tail sheath. These observations are consistent with the final steps of assembly involving components of the contractile tail (43). Likewise, the observation that the expression of the endolysin gene is significantly higher at 40 min compared with 25 min matches expectations as the terminal stage of phage infection involves lysis of the host cell.

**Fig 5:**
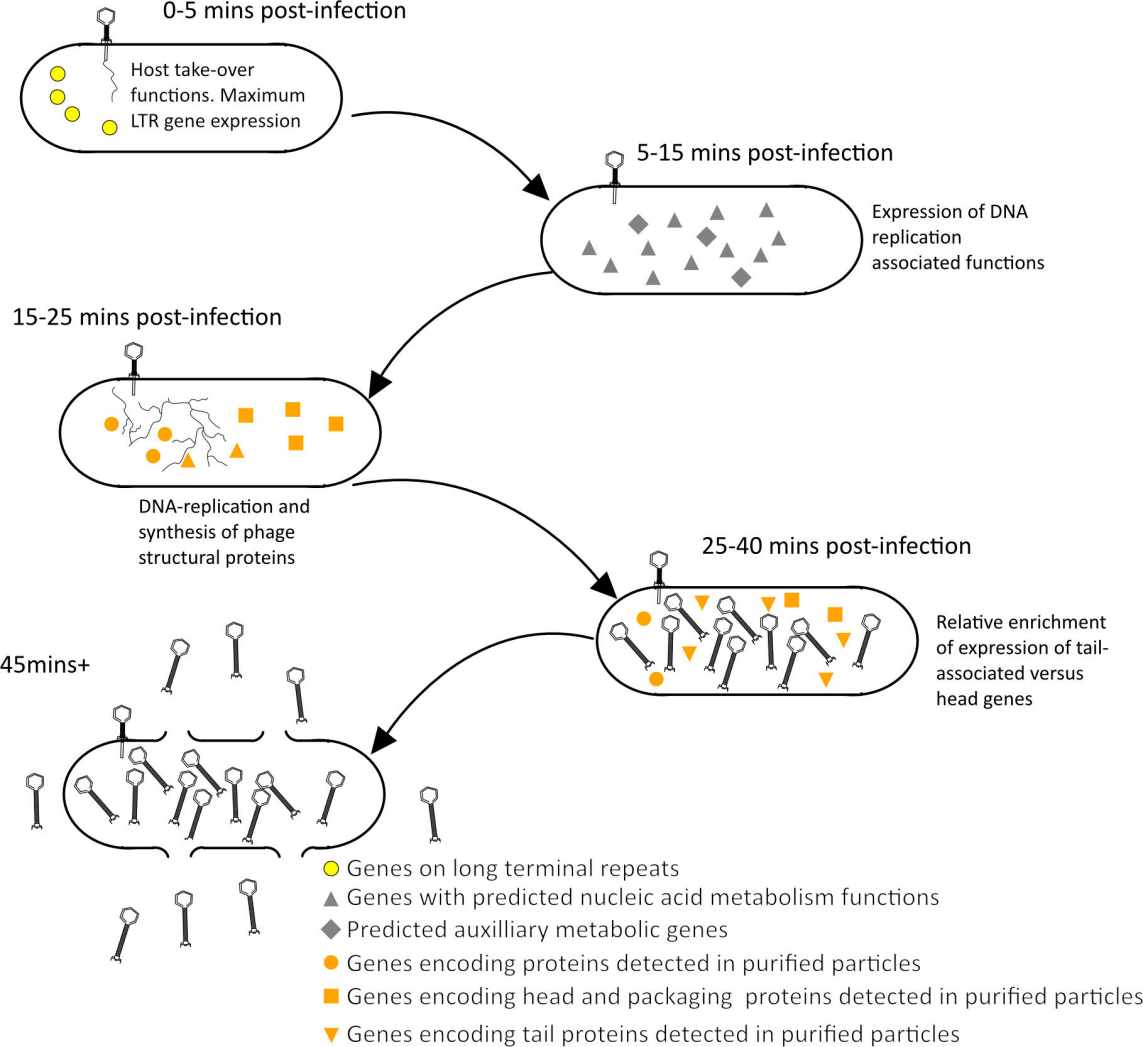
Schematic representation of phage CKA15 infection program. The Illumina-based RNA-seq results revealed a differential abundance of genes with different functions at various time points post-infection. The highest level of expression of predicted host takeover functions and genes on the long terminal repeats (LTR) occurs at 0–5 min post-infection. At 5-15 min post-infection, the highest level of expression of genes with predicted nucleotide metabolism functions was observed. At 15–25 min post-infection, structure and assembly genes are expressed. At 25–40 min post-infection, there is a higher level of expression of tail-associated genes compared with 25 min post-infection.

Therefore, based on the Illumina-based RNA-seq data of phage CKA15-infected *Lm*19111, we discerned a gene expression pattern in which genes with different functional categories are expressed at different times post-infection. The LTR genes were maximally expressed at 5 min among the time points tested, nucleotide and nucleic acid metabolism genes were maximally expressed at 15 min, and the expression of genes encoding structural proteins was highest at 25 and 40 min (Fig. 5).

### Homologs of phage-encoded sigma and anti-sigma factors exhibit trends toward maximum expression in the middle infection stage

We observed an increase in the expression of CKA15_135, which shares homology to an anti-sigma factor encoded by the *Staphylococcus aureus* phage G1 (44, 45), of more than 7-fold increase in mean TPM at 15 versus 5 min. However, the difference does not reach statistical significance (Fig. S3A). A putative phage-encoded RNA-polymerase sigma factor was likewise more abundant at 15 and 25 min compared with 5 min, which is consistent with it having a role in mediating the switch in transcription to late genes (Fig. S3B). To further elucidate potential mechanisms underlying the switch in phage gene expression between early and late infection stages, we identified phage transcriptional boundaries using ONT-cappable-seq.

### Elucidation of phage transcriptional boundaries using ONT-cappable-seq

Following a similar approach described by Leskinen et al. (11), we grouped genes as immediate early, early, middle, or late, depending on whether they reached maximal expression at 5, 15, 25, or 40 min. We divided the promoters upstream of these genes into similar categories and performed motif analysis using MEME (46). Then, we used MAST (47) to search for promoter motifs that the ONT-cappable-seq pipeline may have missed due to low read numbers mapped to certain parts of the phage genome.

### Early phage genes are downstream of DNA elements resembling SigA-activated promoters with an AT-rich UP element

**Fig 6:**
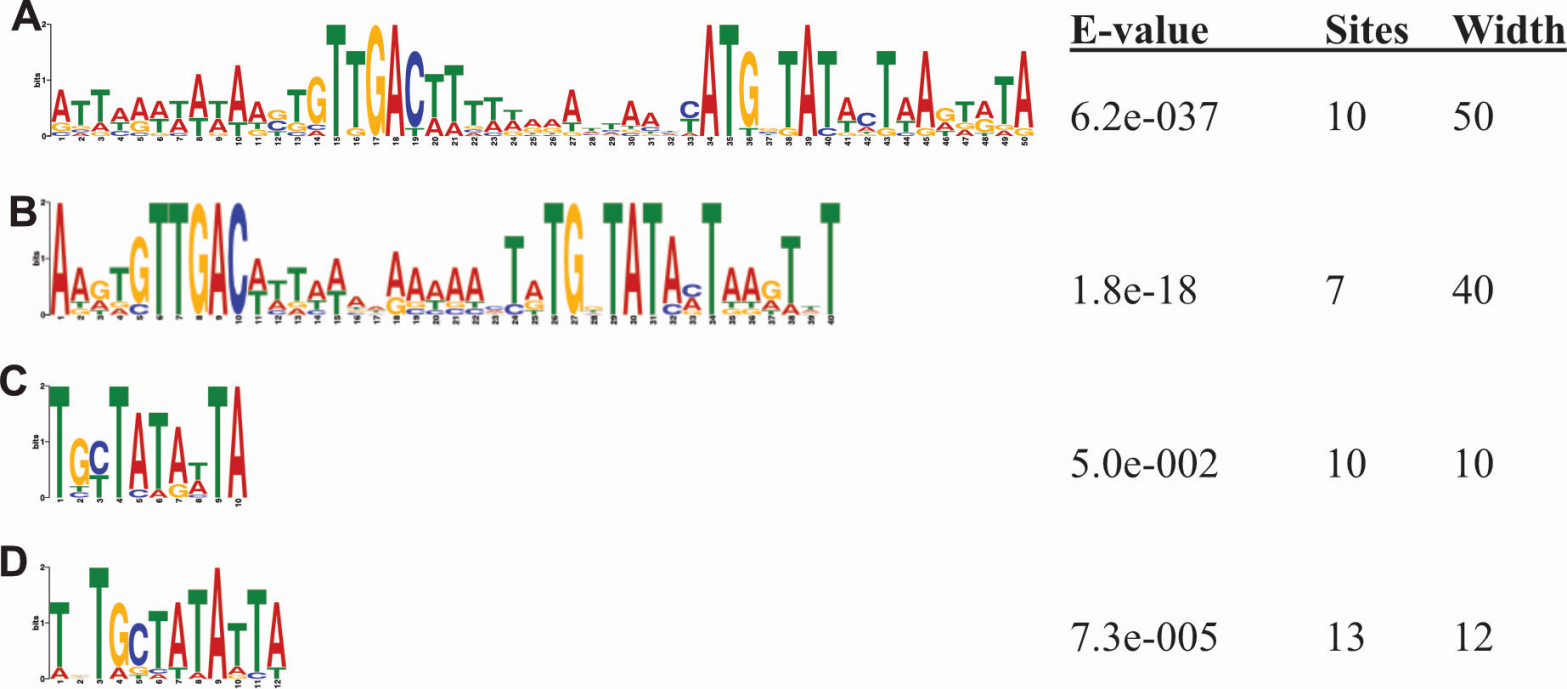
Phage CKA15 promoter motifs. Motifs were identified using MEME by grouping the TSSs upstream of genes with maximal expression at 5 min (**A**), 25 min (**C**), and 40 min (**D**). A motif from the promoters upstream of the seven most transcriptionally active regions at 5 min post-infection was also detected (**B**).

MEME analysis of transcriptional start sites upstream of genes that had maximal expression at 5 min post-infection revealed a motif starting at position −16 and extending to position −5 with the consensus sequence ATGBTATWCTW and the most common sequence being ATGxTATACTTAA. In addition, there is a highly conserved element from −35 to −26 with the sequence TTGACTT. Furthermore, a conserved AT-rich sequence is upstream of the −35 motif (Fig. 6A).

A reverse motif search using MAST with the motif identified from the TSSs upstream genes that had maximal expression at 5 min post-infection revealed 28 additional putative promoters that were missed by the automatic pipeline but that met our inclusion criteria to consider potential promoters. Among the five genes with greater than 1% of the total transcripts at 5 min post-infection and that had discernible promoters, the promoters for three—those upstream of CKA15_059, CKA15_191, and CKA15_194—were identified using reverse motif search. A follow-up motif analysis based on these top five strongest promoters at 5 min post-infection, as well as two promoters driving high levels of transcription from two unannotated regions of the genome close to loci encoding tRNA genes, revealed that these promoters all had a TATAMT sequence and a highly conserved TTGAC sequence 15 bases further upstream. Therefore, the DNA elements upstream of the most highly transcribed phage loci in early infection bear a strong resemblance to the housekeeping σ^A^ consensus promoter sequence from *Bacillus subtilis* (48). However, they lack the AT-rich UP element identified for other early CKA15 genes. Consequently, we identified two potential classes of promoters resembling σ^A^-regulated promoters, of which one was associated with very high levels of expression (>1% TPM) (Fig. 6B).

### Late phage genes are downstream of DNA elements resembling an extended −10 element of SigA-activated promoters but lack the consensus −35 element of SigA-activated promoters

Regarding the promoter motifs upstream of genes with maximum expression at the other time points, no motifs with e-values below 0.05 were identified for the 15 min post-infection time point. A similar motif was identified for the 25- and 40-min time points. The 25-min post-infection time point motif was TGYTATAWTA, based on 11 sequences (Fig. 6C). A search with 17 sequences for the 40 min time point revealed a TDTGCTATATTA consensus sequence, with the most common sequences for the two time points sharing the motif TGCTATATTA (Fig. 6D). The e-value for the 25-min time point motif was 0.05, at the inclusion threshold. In contrast, for the 40 min time point, it was 7.3 × 10^−5^. Considering the similarity between the two motifs and the lower e-value associated with the motif identified from TSSs related to the late genes, we performed MAST analysis with the latter. Thus, we detected an additional seven promoter sites. Among these were putative promoter sites upstream of the genes encoding endolysin and major capsid protein. For the latter, we detected both an early and late promoter motif, consistent with the observed elevated levels of expression of this gene throughout infection.

The observation of distinct phage promoter motifs corresponding to maximal expression at 5 and 40 min post-infection suggests that the switch from early to late gene expression is mediated by a switch in promoter recognition by the host RNA polymerase. We also observed that the transcript abundances of predicted phage-encoded sigma and anti-sigma factor genes reach maximal mean TPM levels at 25 and 15 min, respectively, which is consistent with these gene products having a role in mediating the switch from early to late genes (Fig. S3).

**Fig 7:**
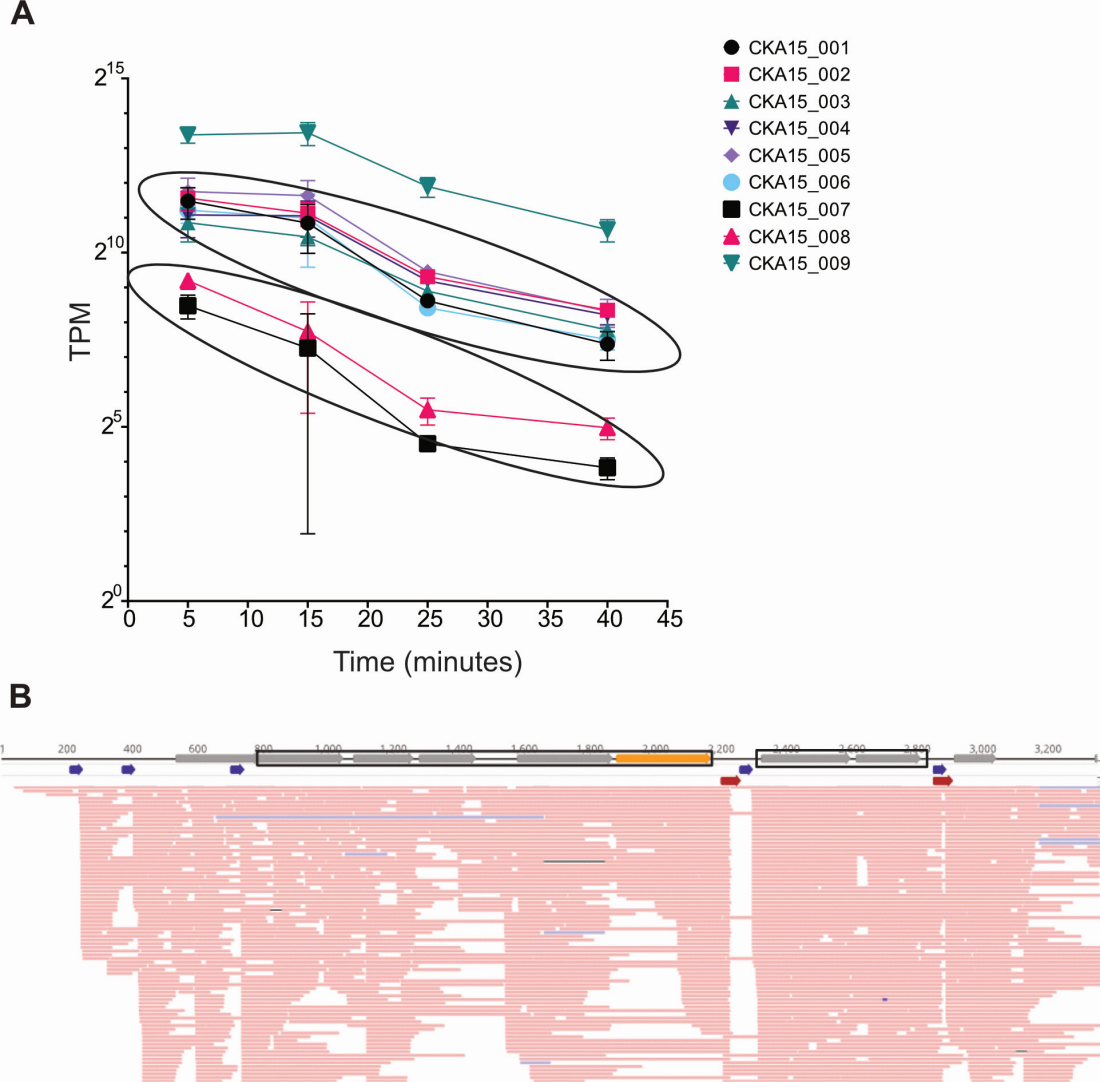
Time-resolved transcript abundance and operon organization of LTR genes. (A) TPM-normalized phage transcript abundance of LTR genes at 5, 15, 25, and 40 min post-infection. Error bars indicate the standard deviation of the mean (*n* = 3). CKA15_002, CKA15_003, CKA15_004, CKA15_005, CKA15_006, and CKA15_007, CKA15_008, respectively, are circled with ellipses to show that these genes are co-transcribed and that the transcript abundance of the co-transcribed genes covaries. (B) Operon organization of CKA15 LTR genes determined by ONT-cappable-seq. Blue and red arrows indicate transcriptional start and stop sites, respectively. CKA15_002, CKA15_003, CKA15_004, CKA15_005, and CKA15_006 are outlined. The orange CDS represents a gene encoding a protein that was detected in purified particles.

In addition to detecting TSSs, ONT-cappable-seq enabled us to detect 66 putative transcription termination sites (TTS), of which 43 are on the top strand and 23 are on the bottom strand. Of these, 13 are predicted by ARnold (49) to be intrinsic terminators. Of note is that neither of the TTS detected on the LTR was identified by ARnold to be intrinsic terminators, despite the apparently efficient transcription termination at these sites (Fig. 7B).

### Identification of co-transcribed phage genes combined with sequence analysis provides insights into potential functions encoded by the phage genome

In the structure and assembly module, the genes encoding tail tube protein and tail assembly chaperone are followed by intrinsic TTS. The latter appears to be part of the same operon as a hypothetical protein-coding gene with locus tag CKA15_114, as it and the tail chaperone genes are flanked by a TSS and a TTS, and there are reads that map to the combined length of the two genes. Since genes with related functions are often co-transcribed, CKA15_114 may have a role in tail assembly. These two genes also have similar expression levels throughout infection, reaching maximum TPM values at 25 min post-infection, with 784 for CKA15_114 and 635 for the tail assembly chaperone gene.

For the identification of transcriptional units, we adapted the approach reported previously (50). For genes to be considered part of the same operon, they must be flanked by a TSS and TTS. Also, ONT-cappable-seq reads encompassing the genes must have been detected. For example, a read spanning positions 633–2,230 on the positive strand (1,598 bp). There is another one spanning 741–2,230 (1,490 bp). These two reads encompass five coding sequences, CKA15_002-006, flanked by a TSS and a TTS. Consistent with these genes being part of the same operon, we observe from the Illumina-based RNA-seq experiment that the abundance of their transcripts is similar throughout infection (Fig. 7B). Note, however, that we do not exclude the possibility that CKA15_001 is also part of the operon encompassing genes CKA15_001-006. Moreover, its belonging to this operon is supported by its transcript abundance covarying with that of genes CKA15_002 to CKA15_006 (Fig. 7A).

Two TSSs were identified upstream of the locus tag CKA15_001, hinting at potential small CDSs (Fig. 7B). There is an ATG triplet 15 bp downstream of the first detected TSS. If this corresponds to a start codon, a CDS encoding a hypothetical protein of only 19 amino acids in length would be present in this frame. There are no apparent start codons downstream of the second TSS detected by the ONT-cappable-seq pipeline that would correspond to a missed CDS. Therefore, the first two TSSs may be tandem promoters driving a relatively high level of expression (close to 3,000 TPM at 5 min) of CKA15_001, in which case, it would be monocistronic, or there may be an additional small CDS upstream of CKA15_001.

Among other genes on the LTR, those with locus tags CKA15_007 and CKA15_008 are also flanked by a detected TSS and TTS. There are dozens of aligned reads that span the length of the two genes, suggesting that these genes are polycistronic and may have related functions. CKA15_015 to CKA15_019 are also flanked by a TSS and TTS, and there is a read encompassing all four genes, indicating that they are likely part of the same operon. All four of these genes are annotated as hypothetical, although HHPred analysis suggested that CKA15_017 is structurally related to pentapeptide repeat proteins from *Clostridium botulinum*, *Mycobacterium* spp., and *Enterococcus faecalis*. These proteins are annotated as DNA gyrase inhibitors or as having roles in fluoroquinolone resistance.

We also identified operons transcribed from the bottom strand that may have functions related to DNA metabolism. CKA15_043 and 042 are likewise flanked by a TSS and TTS and encompassed by reads detected in the ONT-cappable-seq experiment. HHPred analysis suggests that CKA15_043 is structurally related to phosphatases. Although reads were also detected that encompass CKA15_042 and a metallophosphatase gene immediately downstream of CKA15_042, we did not detect any that encompass all three genes. Still, given their proximity on the genome to a metallophosphatase gene, the HHPred prediction, and the presence of a gene encoding a Zn finger-containing domain protein (CKA15_039) for which evidence from the ONT-cappable-seq data exists that it is co-transcribed with the metallophosphatase gene, it is possible that CKA15_043 is a phosphatase with a role in DNA metabolism. Loci CKA15_039 to CKA15_043 likewise had similar expression levels throughout infection, with TPM values peaking at 15 min post-infection for all but CKA15_043, which had slightly higher expression at 5 min. At 15 min, the average TPM values for these four genes ranged from 451 to 848.

Another operon transcribed in the reverse direction includes the genes with locus tags CKA15_052, 053, and 054. HHpred analysis of CKA15_052 and CKA15_054 suggests that these genes may encode a GTP pyrophosphokinase/hydrolase, alarmone synthetase/hydrolase with a role in the stringent response, and a transcriptional regulator with a possible role in quorum sensing inhibition, respectively. The putative transcriptional regulator is structurally similar to Prx, whose structure is complex with the quorum sensing transcriptional regulator from *Streptococcus pyogenes*, ComR, has been solved (51).

Therefore, analyzing the phage genome transcriptional boundaries in light of the time-resolved transcript abundance provided insights into the phage infection program. Integrating the HHPred analysis with knowledge of the timing of expression and operon organization also enabled us to propose potential functions for genes that encode no characterized homologs.

### Host transcriptional landscape during infection by CKA15

Shortly after infection of *Lm*19111 by phage CKA15, the host undergoes profound transcriptional changes. Five minutes after infection, 206 upregulated and 152 downregulated host genes can be observed. Compared with t0, at 15, 25, and 40 min post-infection, there are 297, 248, and 258 upregulated genes and 247, 259, and 212 downregulated genes, with thresholds of *P*adj < 0.001 and |L2FC| ≥ 2. Despite the roughly equal proportion of up- and downregulated host genes at all time points following infection, transcript read mapping follows a pattern of progressively fewer transcripts mapped to the host and more mapped to the phage genome as the infection progresses (Fig. 1B).

Among the upregulated genes 5 min post-infection are many genes encoding proteins with functions related to propanediol catabolism. BlastP analysis of the top 11 most highly upregulated genes showed that 10 of them have homologs annotated as having functions related to propanediol metabolism, including genes encoding PduA, PduB, PduD, PduJ, PduK, PduM, and PduN. Among these are structural components of propanediol utilization bacterial microcompartments.

Genes encoding products responsible for both aerobic (*cob*) and anaerobic (*cbi*) corrin (“core” of cobalamin) ring synthesis are significantly upregulated. In *Listeria* spp., the genes responsible for cobalamin biosynthesis and ethanolamine and propanediol utilization reside in a region of the chromosome called the cobalamin-dependent gene cluster (52). Cobalamin is involved in various bacterial cellular processes, including metabolism and transcriptional and post-transcriptional gene regulation. Several of the highly upregulated genes are in the *pdu* operon, including *pduA*, *pduB*, *pduC*, *pduD*, *pduE*, and *pduL*. The pairwise comparison between uninfected bacteria and 5 min post-infection shows that genes in this operon are upregulated after infection with L2FC values ranging from 8.3 to 12.7. These genes are involved in the cobalamin-dependent catabolism of 1,2-propanediol. Among these are genes that encode components of bacterial microcompartments, including *pduA* and *pduB*. The genes *pduCDE* encode a coenzyme B12-dependent propanediol dehydratase.

At 5 min post-infection, *tagO* is significantly downregulated (L2FC ~ 2.2). This gene encodes a putative undecaprenyl-phosphate N-acetylglucosaminyl 1-phosphate transferase, the enzyme that catalyzes the first step in the biosynthesis of teichoic acids. In 1/2a serotype strains of *L. monocytogenes*, which includes *Lm*19111, wall teichoic acids are rhamnosylated (53). The catabolism of rhamnose yields propanediol, the metabolism of which requires genes encoded on the CDGC, including *pduABCDE*. As noted above, these genes are among the most highly upregulated following phage infection.

Eugster and Loessner (20) reported that deletion of both *L. monocytogenes tagO* homologs is necessary for a reduction in WTA to be observed. In our data set, we see downregulation of only one *tagO* homolog despite two being present in the genome of *Lm*19111. However, alongside the reduction in *tagO_2*, we also observed a reduction in *tagG*, *tagT,* and *tagA* (L2FC −1, −1.41, and −1.43), although the magnitude of these changes does not reach the arbitrary threshold of |L2FC| ≥ 2 that we set. However, for *tagT* and *tagA,* the padj values are below the stringent threshold of 10^−3^. Consequently, the combined effect of these somewhat modest changes in gene expression may lead to a phenotypically significant reduction in WTA biosynthesis and a surplus of intracellular rhamnose, as this sugar is diverted from the glycosylation of WTA.

**Fig 8:**
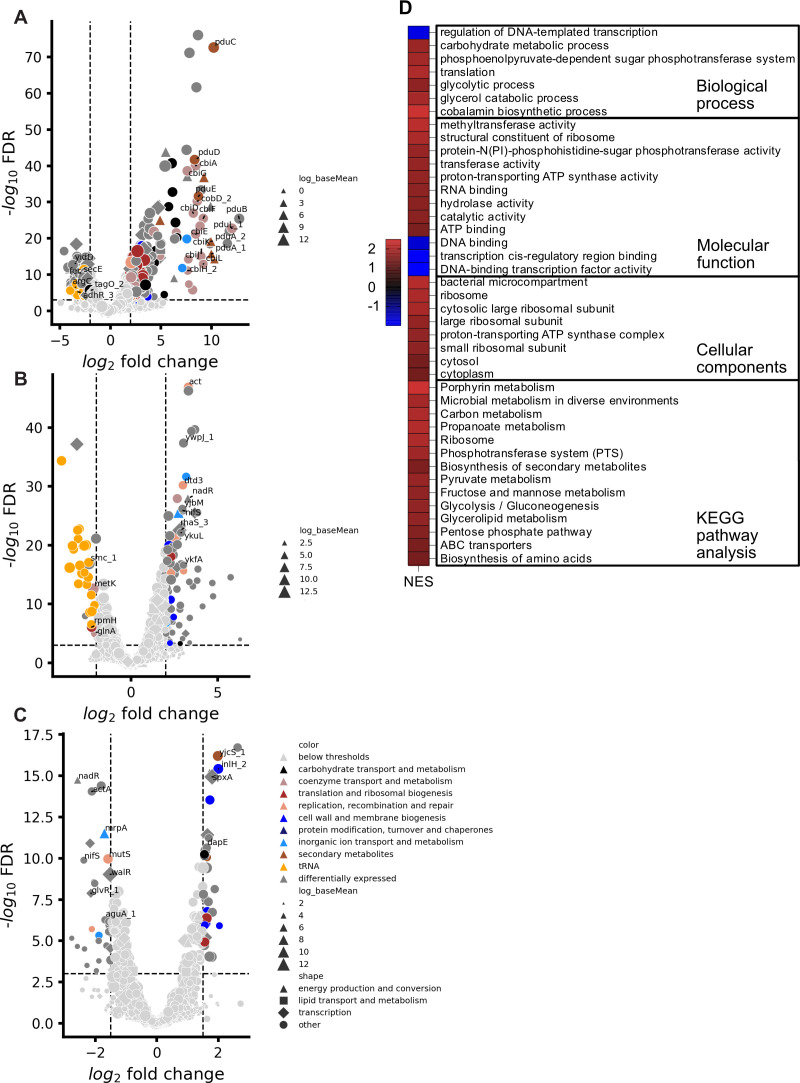
Host transcriptomic state during infection. (**A, B, C**) Pairwise comparison of host transcript abundance between 0 and 5, 5 and 25, and 25 and 40 min post-infection, respectively. The legends for panels A, B, and C are the same except for log_base mean. The functional categories are COGs. The |L2FC| thresholds are 2 for panels A and B and 1.5 for panel C. (**D**) Gene set enrichment analysis comparing host gene expression in uninfected control with 5 min post-infection based on GO and KO terms.

Although most of the changes in host transcription levels were evident at 5 min post-infection (Fig. 8A), some changes were observed between 5 and 25 min post-infection as well as 5 and 40 min post-infection (Fig. 8B and C). At 25 min, genes with a cluster of orthologous group (COG) designation “replication, recombination, and repair” were upregulated compared with 5 min (Fig. 8B). At 40 min post-infection, there were some genes with functions related to cell envelope biogenesis, including a probable *dapE* homolog, that were expressed at higher levels compared with 5 min, although the magnitude of the differences was modest (Fig. 8C).

Since most of the transcriptional changes in *Lm*19111 were evident at 5 min post-infection, we focused our gene ontology (GO) and KEGG pathway analysis on the 5 min versus uninfected pairwise comparison. GO terms from InterProScan and KEGG Orthology (KO) terms were mapped to *Lm*19111 genes to perform gene set enrichment analysis (GSEA). The GO GSEA revealed translation, cobalamin biosynthesis, and carbohydrate metabolism among the upregulated processes and regulation of DNA-templated transcription as the only downregulated biological process (Fig. 8D). Molecular functions that were upregulated include methyltransferase, ATP synthase, ATP binding, RNA binding, and structural constituent of ribosome. Downregulated molecular functions include DNA binding, transcription *cis*-regulatory region binding, and DNA-binding transcription factor activity. The cellular components hierarchy GSEA shows enrichment of ribosome, bacterial microcompartment, and proton-transporting ATP synthase complex. The KEGG pathway analysis GSEA shows increased pathways associated with the *Listeria* cobalamin-dependent gene cluster, including porphyrin metabolism, propanoate metabolism, and biosynthesis of secondary metabolites. Other pathways were upregulated, including carbon metabolism, pyruvate metabolism, phosphotransferase system, and several others.

## DISCUSSION

In the present work, we characterized a phage belonging to the genus *Pecentumvirus* by analyzing mature purified phage particles by ESI-MS/MS. Moreover, we applied two different RNA-seq techniques to elucidate the phage operon structure and time-resolved transcript abundance of the phage and its host during infection. To our knowledge, this is the first report of a transcriptomic analysis of a lytic *Listeria* phage. Due to its similarity to other members of the genus, much of what we learned from this study of CKA15 is likely generalizable to other P100-like phages.

A recent study by Fernandez et al. (54) reported comparative genomic analysis of 21 phages in the genus *Pecentumvirus*. Among the 21 was AG20, which shares >99% intergenome identity with CKA15 (9). Fernandez et al. reported that most of the genus’ gene gain and loss events occur within 20 k bp of the genome’s end. In the present work, we observed that the ends of the genome of CKA15 primarily encode genes that have the highest levels of expression at 5 min post-infection among the time points we tested. These observations are consistent with changes in the repertoire of host-takeover functions being an important driver of P100-like phage adaptation to their hosts (54). Moreover, based on our time-resolved RNAseq experiment and given the assumption that phage genes that are maximally expressed at 5 min are involved in host takeover, the genome regions encoding this function extend beyond the long terminal repeats of the phage.

Klumpp et al. (16) performed genomic and proteomic characterization of A511, which is a well-characterized member of the genus *Pecentumvirus*. They used MALDI-TOF to identify 16 proteins in the SDS-PAGE gel of purified A511 particles. For our part, we detected 29 phage proteins, including the putative endolysin. Although Klumpp et al. excised individual bands, we cut out the entire lane, including the dye front. As noted by Klumpp et al., compared with other SPO1-like phages, including SPO1 and phage K, in A511, the location of the endolysin gene is in an unusual genomic location, namely between the portal protein and TerL (terminase large subunit) encoding genes. This situation is comparable with CKA15. Moreover, we detected two phage proteins in purified phage particles in this genomic location, one of which is the endolysin and the other a gene (locus tag CKA15_094), which was previously annotated as a hypothetical protein but to which Pharokka assigned a Phrogg identifier corresponding to head and DNA packaging function. The latter gene also had the highest mean expression (TPM) at 40 min post-infection, although the mean TPM values for it between the 25 and 40 min time points post-infection were similar.

Given the high sensitivity of ESI-MS/MS, we cannot conclude that all the proteins detected in the mature purified particles, encoded by genes outside of the structure and assembly module, represent genuine particle-associated proteins. However, this possibility cannot be excluded. Some detected proteins may be co-injected with the phage genetic material at the start of infection. Examples in the literature include Mu protein N, which forms a complex with the phage Mu genomic DNA (55) as well as the virion-associated RNA polymerases of phages N4 (32) and phiKZ (56). Besides the detection of the putative CKA15 endolysin in mature particles purified by two rounds of CsCl gradient centrifugation, further evidence for its potential presence associated with mature particles is provided by the zymograms we performed in parallel with the SDS-PAGE, using the same suspension. The only clearings identified can be accounted for by a polypeptide with the molecular weight of the full-length endolysin and the N-terminal enzymatically active domain of the protein. The latter may be a degradation byproduct that retains some PG-degrading activity without the C-terminal cell wall binding domain. However, alternate translation of the protein cannot be excluded.

Our proteogenomic experiment consistently detected several other homologs of A511 proteins whose localization was previously confirmed by immunogold labeling and antibody cross-linking experiments, including adsorption-associated proteins (25). Experiments by Habann et al. (25) identified two proteins in the tail machinery of phage A511 that play roles in adsorption. In A511, these proteins are annotated as gp108 (receptor binding protein in CKA15) and gp98 (spike protein in CKA15). We detected the CKA15 homologs of both proteins in the proteogenomic experiment with purified phage particles. The baseplate “spike” seems to consist of two proteins, which in CKA15 are encoded by genes with locus tags CKA15_118 and CKA15_119. The homolog of CKA15_118 in A511 (gp98) plays a role in adsorption, which was established using adsorption assays done in the presence of anti-sera against various components of the A511 tail machinery. Haban et al. (25) also reported that two of the tail proteins—the TMP and gp98—in A511 have domains with predicted enzymatic activity and suggested that these may play a role in cell wall puncturing following adsorption to host cells.

Early work investigating SPO1 infection of *B. subtilis* found that approximately 70% of the cellular RNA originated from SPO1 by the end of infection (10). More recently, transcriptomic studies of infection of *S. aureus* by lytic phages related to phage K (11, 23, 45). Transcriptomic studies in *Kayvirus* members, of which phage K is the type species, suggest a range from approximately 40% (57) to 70% (39) of transcripts mapping to the phage by the end of infection may be typical for *Herelleviridae* phages. Our transcriptomic study of CKA15 likewise falls in this range. However, heterogeneity in transcriptional activity seems to be the norm, with certain regions of the phage genome accounting for a disproportionate percentage of mapped reads.

The genome of CKA15 shows a high degree of heterogeneity in transcriptional activity, as assessed by the number of reads aligned to the phage genome per position. Three regions appear to be non-coding but had large numbers of reads aligned to them in the time-resolved RNA-seq experiment compared with the rest of the genome. Our group initially annotated one of the regions with the highest number of reads aligned and spanning positions 26,236–26,727, as containing three protein-coding sequences, based on the assumption of a high density of protein-coding sequences in phage genomes. However, subsequent BlastP analyses revealed no homologs to these predicted proteins. MAST analysis using the identified promoter motif associated with maximal expression at 5 min post-infection detected promoters upstream of the three highly transcriptionally active regions. The alignment of reads per position is consistent with the expression levels of the most active region progressively increasing until late infection. For the other two regions, which have the second and third most reads aligned per nucleotide position in the phage genome, the deepest mean sequencing coverage was observed at 15 and 25 min post-infection.

We initially hypothesized that these regions may encode small interfering RNA species that play a role in translational silencing and degradation of host transcripts via the action of specific ribonucleases. Considering that many sRNAs have been identified in *Listeria* spp (58, 59), it is a sensible inference that the cellular physiology of this bacterium may be susceptible to modulation by phage-encoded sRNAs. However, the absence of canonical intrinsic terminator motifs at the 3′ ends of the transcripts detected by ONT-cappable-seq and the lack of UCCC motifs argue against the explanation that the three highly transcribed regions encode siRNAs (58). On the other hand, the presence of the UCCC motifs in bacterial siRNA could mean that in the context of phages, these motifs could be selected against for reasons of orthogonality.

Another possible explanation is that the highly abundant RNA species in question may be Y RNAs, which are a species of cytoplasmic ncRNAs that were first identified in eukaryotes but have since been identified bioinformatically in diverse bacterial species as well as mycobacteriophages (60). Available information on the role of Y RNAs in prokaryotes suggests that they are tRNA mimics that play a role in RNA metabolism and quality control of non-coding RNAs (61). HHPred indicated a potential role of CKA15_055 in RNA binding, with the highest confidence match being with a Ro sixty-related (Rsr) protein encoded by *Deinococcus radiodurans*. This locus is transcribed within 2 kbp downstream of the three highly transcriptionally active regions of the phage genome in the same direction as these regions. These observations are consistent with CKA15_055 encoding an Rsr protein and one or more of the putative ncRNAs being Y RNAs, examples of which include parts of RNA-protein complexes (62).

The Rsr homolog in *D. radiodurans* was shown to form an RNA-protein complex with the 3′ → 5′ exonuclease PNPase via a scaffolding Y RNA and enhance the ability of PNPase to degrade structured RNAs with a disordered 3′ end (62). CKA15_055 may be playing a similar role in CKA15-infected bacteria, contributing to changing the population of RNA species by forming complexes with either host or phage-encoded ribonucleases scaffolded by the highly transcribed Y RNA species encoded by the phage. An alternative explanation is that CKA15_055 and the putative Y RNAs play a role in the quality control of rRNA, which is another function that has been associated with Y RNAs and Rsr homologs (61). This explanation would be consistent with the observed enrichment of bacterial genes related to translation following infection and the need for the phage to optimize the cellular environment for efficient translation of phage polypeptides. Verifying which of the above explanations for these highly abundant RNA species corresponds to reality—whether siRNAs, Y RNAs, or whether the abundant RNA species are protein-coding—as well as what role they play in the phage replicative cycle will require dedicated biochemical analysis.

ONT-cappable-seq is a powerful transcriptomic technique combining primary transcript enrichment and long-read sequencing. Applied to *Lm*19111 infected by CKA15, it enabled us to identify transcription start and termination sites across the phage genome. Combining it with motif analysis using MEME and reverse motif searching using MAST, we identified putative promoter sites across the genome of CKA15.

The combination of time-resolved Illumina-based RNA-seq, motif analysis of TSSs identified by ONT-cappable-seq, and HHPred enabled us to predict a regulatory mechanism underlying the switch from early to late promoters in phage CKA15. CKA15_135 has been annotated as an anti-sigma factor in related phages. HHPred analysis revealed that it is closely related to an anti-sigma factor encoded by the *S. aureus* phage G1 that interacts directly with σ^A^ and alters promoter specificity of the RNA polymerase holoenzyme by preventing the interaction of σ^A^ with AT-rich motifs present in some σ^A^-recognized promoters upstream of the −35-consensus sequence (45). Similar AT-rich regions upstream of promoters driving expression of genes that had maximal expression at 5 min post-infection suggest that CKA15_135 may mediate the shut-down of immediate early genes. Accordingly, the highest mean TPM values for CKA15_135 were at 15 min post-infection. Notably, although the motif identified from all TSS upstream of genes with maximum expression at 5 min post-infection had an AT-rich region upstream of the −35 TTGACCT element, this was not observed for the motif obtained from the top seven strongest promoters at 5 min post-infection, nor for the promoter likely driving transcription from region 26,727 → 26,236, which showed progressively increasing transcriptional activity until the end of infection.

Regarding the host transcriptome during infection, one of the observations from our analysis is that the host transcriptional landscape is drastically altered within a short period following infection. This is similar to what has been reported for *S. aureus* infected by a *Kayvirus* (63). Our GO analysis revealed the enrichment of host translation-related functions at the levels of biological process, molecular function, and cellular component. The only downregulated host functions were those related to transcriptional regulation and DNA binding. Our KEGG pathway analyses revealed changes consistent with propanediol utilization and cobalamin biosynthesis. Enrichment of porphyrin and propanoate metabolism, which are enriched in the KEGG pathway GSEA, and methyl transferase activity, enriched in the molecular function GSEA pathway analysis, is consistent with the upregulation of synthesis of methylcobalamin. In the cellular component GSEA, the enrichment of bacterial microcompartment cellular component is also consistent with propanediol utilization since bacterial microcompartments for propanediol utilization have been demonstrated in *L. monocytogenes* (64).

Other groups have reported upregulation of the *L. monocytogenes* cobalamin-dependent gene cluster following high-pressure processing, cultivation in vacuum-packaged cold smoked salmon, and co-cultivation with bacteria associated with cheese rinds, namely isolates of *Psychrobacter* and *Brevibacterium* (65–67). Furthermore, A. R. Varadarajan et al. (68) used a proteogenomic approach to identify differentially abundant proteins in *L. monocytogenes* strains ScottA and EGD-e under conditions of low pH, high osmolarity, and bile salts. They found that EGD-e, like *Lm*19111, belongs to serotype 1/2a and displayed an increased abundance of numerous proteins encoded on the CDGC in the presence of bile salts compared with cultivation in 1% buffered peptone water. L. Vásquez et al. (69) also showed that *cbiP*, which is part of the CDGR and catalyzes the synthesis of adenosyl corbamide, plays a role in tolerance to copper and low temperatures. Taken together with our data showing upregulation of cobalamin biosynthesis following phage infection, the preceding observations suggest that upregulation of the CDGC in *L. monocytogenes* occurs following exposure to some abiotic and biotic stresses and may be part of a general stress response.

In conclusion, we have performed a comprehensive multi-omics characterization of the strictly lytic *Listeria* phage CKA15. Our approach involved proteogenomic detection of presumptive mature phage particle-associated proteins, identification of transcription start and termination sites using ONT-cappable-seq, and time-resolved transcriptomics of both host and phage transcripts using Illumina-based RNAseq. We observed 29 presumptive phage particle-associated proteins, two distinct phage promoter motifs that are likely the basis for the switch from early to late phage gene expression, and large-scale transcriptional reprogramming of the host evident early in infection. This work provides a systems-level understanding of lytic phage infection of *L. monocytogenes* by a P100-like phage, and it serves as a point of departure for generating hypotheses to be tested using a combination of biochemical, genetic, and microscopic techniques.

## MATERIALS AND METHODS

### Cultivation of bacteria and phage

*L. monocytogenes* ATCC19111 (*Lm*19111) 25%(vol/vol) glycerol stocks stored at −80°C were streaked onto brain heart infusion (BHI) agar and incubated at 37°C overnight. Individual colonies were inoculated into BHI and incubated at 30°C with agitation (180 rpm). The following day, overnight cultures were diluted 20-fold and grown to the exponential phase under the same conditions.

For phage propagation, a one-step growth curve, and infection assays, CaCl_2_ was added to the BHI to a concentration of 2 mM (cBHI).

Phage CKA15 was propagated using *Lm*19111 as the host. *Lm*19111 was grown at 30°C in cBHI until an OD_600_ of 0.3–0.5 was reached, and CKA15 was added to a titer of 5–6 log_10_ PFU/mL. Then, the agitation rate was reduced from 180 to 130 rpm, and the culture was incubated overnight. The next day, the lysed culture was centrifuged at 7,000 × *g* for 20 min, and the supernatant was filtered through 0.2 µm PES bottle-top filters. The filtrate was titrated using CM buffer (5 mM CaCl_2_, 21 mM MgSO_4_, 6 mM Tris-HCl, pH 7.2) as the diluent, and spotting on lawns of Lm19111, which were prepared using the soft overlay method (70), with 0.5% agar BHI as the overlay medium.

### CKA15 one-step growth curve

The one-step growth curve (OSGC) protocol for CKA15 was adapted from Kropinski (71). Briefly, cultures in biological triplicate were grown to an OD_600_ of ~0.8 in cBHI. The phage stock suspension contained 1.5 × 10^7^ PFU/mL. The volume of the stock suspension that was added to 9.9 mL of mid-log (OD_600_ of 0.8) *L. monocytogenes* in cBHI was 0.1 mL. Thus, the phage was added to the adsorption tube to a concentration of 1.5 × 10^5^ PFU/mL. Time zero was set as the moment that the phage was added to the adsorption tube. After 3 min, 0.1 mL from the adsorption tube was transferred to 9.9 mL of fresh cBHI (tube A), and then 1 mL from tube A was transferred to 9 mL of fresh cBHI (tube B).

For the plaque assay, 20 µL aliquots were collected from tubes A and B and plated onto a soft agar overlay containing *Lm*19111 (70).

### CKA15 proteogenomics

Prior to the separation of phage polypeptides on an 8% SDS-PAGE gel, crude phage lysate was concentrated using a 100 kDa MWCO Amicon protein concentrator (15 mL working volume, Millipore) according to the instructions from the supplier, with the following changes. The membranes were pre-treated with PBS containing 1% BSA for 1 hour, and following ultra-filtration of crude lysate, sonicated in a water bath (Fisherbrand CPX2800) on high for 3 min, to maximize recovery (72). The retentate was then purified with two rounds of CsCl gradient centrifugation (73) using a Beckman Coulter Optima XPN centrifuge and dialyzed against two changes of CM buffer with 12 kDa MWCO dialysis tubing. The resulting suspension was concentrated with a 0.5 mL working volume Amicon protein concentrator (10 kDa MWCO, Millipore). In this step, the membrane was not pre-treated with BSA but was again sonicated with a Fisherbrand sonicating water bath (CPX2800) after centrifugation to maximize recovery.

The resulting suspension with a titer of 2 × 10^12^ PFU/mL was loaded onto an 8% SDS-PAGE gel (2 × 10^10^ PFU/lane) and ran at 100 V alongside a dual-color protein standard. The gel was then stained with Coomassie blue using the fast stain and destain protocol (74) and imaged using the ChemiDoc XRS + GelDoc system (Bio-Rad).

The entire lane in which the phage particles were loaded, including the dye front, was excised with a lancet, cut into seven slices of approximately 1 cm width each, and placed into low protein-binding 2 mL microfuge tubes.

All glassware used to prepare reagents for mass spectrometry was rinsed with boiling Milli-Q water, then with 100% isopropanol, and then dried in a BSC. Gel slices were destained by covering them with 25 NH_4_HCO_3_/50% acetonitrile (ACN) solution, vortexing periodically for 10 min, centrifuging, discarding the supernatant, and repeating until the stain was removed. The gel slices were then covered with 100% ACN, incubated for 5 min with occasional mixing and centrifuged, so that the supernatant could be discarded. This was repeated until the gel slices were white and shrunken. The gel slices were then covered with 10 mM dithiothreitol (DTT) and incubated at 50°C for 30 min. After that, the gel slices were washed with 100% ACN, covered with 55 mM iodoacetamide, and incubated at room temperature in the dark for 30 min. Subsequently, the reduced and alkylated gel slices were washed with 50 mM NH_4_HCO_3_ for 15 min with occasional vortexing, treated with ACN to shrink the particles, and dried with a Speedvac (Eppendorf Vacfuge Plus) for 20 min.

The dried gel slices were digested overnight at 37°C with 0.67 µg of sequencing-grade trypsin in 50 mM NH_4_HCO_3_. The following day, peptides were extracted by adding 50 µL of H_2_O to each tube and sonicating the gel slices for 10 min. After vortexing and centrifuging the sample, the supernatant was transferred to siliconized microfuge tubes. A volume of 75 µL 5% formic acid (FA) in 50% ACN was added to the gel slices, followed by 2 min of vortexing, centrifugation, and 5 min of sonication. Then, the supernatant was pooled with the tryptic peptides from the previous step. Subsequently, the last step was repeated, except the sonication was omitted.

After cleaning the peptides with C18 filters, tryptic peptides were analyzed on an Orbitrap Exploris 240 mass spectrometer with a 120-min gradient and 300 nL/min flow rate.

The raw data were analyzed using MaxQuant (2.1.0.0) (75) with the following search parameters: minimum peptides: 2, Match between runs: yes, Fractions: yes, and Label-free quantification: none. The search file used was a FASTA file produced using EMBOSS sixpack (76), which contained all possible proteins greater than 30 amino acids in length translated from the genome of CKA15 in all six frames.

### RNA extraction from the phage-infected bacteria

*Lm*19111 were grown in BHI + 2 mM CaCl_2_ (cBHI) at 30°C at 130 rpm until an OD_600_ of close to 0.5 was reached, corresponding to a cell density of 5 × 10^8^ CFU/mL. At this point, the agitation rate was reduced to 130 rpm. Cultures of *Lm*19111 were infected by phage CKA15 in biological triplicate at a multiplicity of infection of approximately five at an OD_600_ of 0.5. The T0 sample was collected before the addition of phage. After phage addition, samples were taken at 5, 15, 25, and 40 min post-phage addition. Four milliliters of culture were mixed with 0.8 mL of stopping solution (5% water-saturated phenol; pH 4.5–5 and 95% ethanol) in 15 conical tubes, mixed by inversion, flash frozen in liquid nitrogen, and stored overnight at −80°C.

The following day, frozen cultures in stopping solution were thawed on ice and centrifuged at 4°C at 4,600 × *g* for 10 min in a Sorvall Legend RT Plus Centrifuge centrifuge (Thermo Fisher Scientific). RNA extraction was done by adapting the protocol developed by E. Villa-Rodríguez et al. (77). Each pellet was dissolved in 0.5 mL lysis buffer (30 mM Tris, 10 mM EDTA, pH 6.2, 15 mg/mL lysozyme). The pellets in lysis buffer were incubated for 30 min at 37°C in a water bath with periodic mixing. Aliquots of 1 mL of TRI reagent and 0.3 mL of CHCl_3_ were added to each dissolved pellet and centrifuged at 15,700 × *g* for 15 min at 4°C in a cooling benchtop centrifuge (Beckman Coulter Microfuge 22R Centrifuge). The aqueous phases were transferred to RNase-free tubes, adding 0.3 mL isopropanol and 0.3 mL 0.6 M NaCl/0.4 M sodium citrate. The resulting suspension was stored at −20°C for 2 hours. After this, the solutions were centrifuged for 20 min at 4°C and 15,700 × *g* to sediment the RNA. The pellets were washed with 0.5 mL ice-cold 70% (vol/vol) ethanol. The nucleic acid pellets were air-dried in a flow hood and resuspended in 50 μL nuclease-free water. The RNA concentration was measured spectrophotometrically. Purified nucleic acid was treated with Turbo DNase as described in the Turbo DNA-free procedure for rigorous DNase treatment, except that 2 U of DNase were added two times at 30-min increments for a total of 4 U per 20 μL reaction and for a total incubation time of 1 hour using a Turbo DNA-free kit (Thermo Scientific; AM1907)

Following DNase treatment, the concentration of RNA was measured using a Qubit RNA High Sensitivity kit (Thermo Scientific; Q32852). The integrity of RNA was measured using an Agilent TapeStation system with a High Sensitivity RNA ScreenTape (Agilent; 5067-5579). For short-read sequencing, RNA-seq cDNA libraries were prepared for the RNA samples using an Illumina Stranded Total RNA prep, Ligation with Ribo-Zero Plus kit (Illumina; 20040525) according to the manufacturer’s instructions. Sequencing was performed at Genomics Core Leuven.

### Illumina-based RNA-seq data analysis

The raw reads from the Illumina-based RNA-seq experiment were processed as follows. Raw reads were first analyzed using FastQC, trimmed with Trimmomatic (SLIDINGWINDOW:4:20), and then analyzed with FastQC again to verify that the quality of the reads improved and that the Illumina adapters were removed. Read mapping was performed using BWA MEM. From this, the SAM format output was converted to a BAM file. SAM tools were used to index and sort the reads. Counting reads aligned to the phage and host genomes was done using featureCounts. Subsequently, a master CSV file containing all the count data for all the samples and all the reads assigned to rRNA was removed. The count data from this table were then FPKM normalized to perform PCA. PCA was performed and plotted in R using ggfortify, ggrepel, and pccomp.

Differential expression analysis was performed using DeSeq2 using non-normalized count data with rRNA reads being removed. Corresponding metadata files were made for each pairwise comparison of interest. For visualization using volcano plots, output CSV files from DeSeq2 were first filtered to separate the phage and host reads. To assign PHROG IDs (78) to phage coding sequences, automatic annotation of CKA15 was performed using Pharokka (79). The output PHROG IDs from Pharokka were mapped to the CDSs previously annotated in CKA15. Volcano plots were made using Python in the seaborn, matplotlib.pyplot, numpy, adjustText, and pandas packages.

The read counts with rRNA reads removed were normalized using Transcript Per Million (TPM) normalization to compare phage transcript abundance across the 4 time points post-infection. After TPM normalization, the phage counts were used to make a global gene expression heatmap and line plots in GraphPad Prism 8.4.3. For the heatmap, the TPM values for each coding sequence were further normalized by min-max normalization such that 0 corresponded to the time point with the lowest mean expression across the three replicates, and 100 corresponded to the highest mean expression.

*Lm*19111 predicted proteins were assigned KEGG ko numbers with eggNOG mapper v2 (80), and Gene Ontology (GO) terms with UniProt. Gene set enrichment analysis (GSEA) was performed with clusterProfiler v3.10.1 (81) in R, using the GSEA function on both KEGG and GO IDs. For GSEA, the gene list was first sorted by log2 fold change values between uninfected and 5 min post-infection treatments, and terms with a BH-adjusted *P*-value < 0.05 were considered significant.

### Mapping the transcriptional landscape using ONT-cappable-seq

The protocol for ONT-cappable-seq has been described previously (50, 82). The steps are described below, but the reader can refer to prior work for more detail (82). RNA samples were collected from 5, 15, 25, and 40 min post-infection and pooled, as described previously (48).

ONT-cappable-seq library preparation involves purification and DNase treatment of total RNA, followed by poly A-tailing and desthiobiotin (DTB)-GTP capping of the RNA. After capping and polyA tailing, the resulting RNA is split into two workflows, one corresponding to the enriched fraction and another to the control. In the enriched workflow, primary transcript enrichment is performed using streptavidin magnetic beads, followed by first-strand cDNA synthesis, selection of full-length cDNAs, second-strand synthesis, PCR barcoding and amplification, and adaptor ligation. The primary transcript enrichment and selection of full-length cDNA by streptavidin enrichment steps are omitted in the control RNA.

As an internal control, spike-in RNA is added, which is produced using the HiScribe T7 High Yield RNA Synthesis kit using a control template to produce a standardized quantity of transcripts with 5` triphosphate ends. RNA spike-in preparation was done as described previously (82).

Analysis of ONT-cappable-seq sequencing data was performed using the in-house automated pipeline, as described by Putzeys et al. (82, 83). The pipeline broadly encompasses three steps: read processing, read mapping, and detection of transcript boundaries (82).

Following the automatic identification of transcription start sites (TSS) and transcription termination sites (TTS) in the phage genome, transcript boundaries were manually curated by visualizing the alignments and boundaries in IGV (84). TSSs were divided according to the timing of maximal expression of the genes downstream of the TSSs, and a motif search was done using MEME (46) with the following parameters: meme sequences.fa -dna -oc . -nostatus -time 14400 -mod zoops -nmotifs 3 -minw 6 -maxw 50 -objfun classic -revcomp -markov_order 0. Subsequently, we used the Motif Alignment and Search Tool (MAST) (47) to identify promoter elements in the genome of CKA15 that the automated ONT-cappable-seq pipeline may have missed. Motifs with e-values less than 10^−5^ were added to the annotation, except when these conditions were met: the promoter would be in the middle of a coding sequence, driving transcription in the opposite direction of the gene and with no evidence from the ONT-cappable-seq data to support an internal promoter driving transcription of antisense transcripts. The command line summary for the MAST search is mast -oc. -nostatus -minseqs 1 -remcorr -ev 10.0 motifs.meme sequences.fa.

## Data Availability

The raw sequencing data from the Illumina-based and ONT-cappable RNAseq experiments were deposited to SRA. The accession numbers for these data are listed in Table S4.

## SUPPLEMENTAL MATERIAL

Supplemental figures and tables msystems.00587-25-s0001.docx Figures S1 to S3; Tables S1 to S4.

ASM does not own the copyrights to Supplemental Material that may be linked to, or accessed through, an article. The authors have granted ASM a non-exclusive, world-wide license to publish the Supplemental Material files. Please contact the corresponding author directly for reuse.
