# Supplementary material for: Multi-omics characterization of a lytic phage targeting Listeria monocytogenes
Source: mSystems. 2025 Jun 25;10(7):e00587-25. doi: 10.1128/msystems.00587-25 (PMC12282133; doi:10.1128/msystems.00587-25)
Supplement: Supplemental figures and tables — Figures S1 to S3; Tables S1 to S4. [file msystems.00587-25-s0001.docx]

**Supplemental**

**Table S1.** Plaque counts from one-step growth curve.

|  | **Plaque counts (PFU, standardized to tube A)** | | | |
| --- | --- | --- | --- | --- |
|  |  |  |  |  |
| Time | R1 | R2 | R3 |  |
| Adsorption control | 4 | 0 | 5 |  |
| 3 | 27 | 26 | 33 |  |
| 18 | 25 | 24 | 30 | A |
| 33 | 30 | 24 | 30 |  |
| 48 | 28 | 21 | 29 |  |
| 58 | 81 | 40 | 38 |  |
| 63 | 270 | 180 | 140 |  |
| 68 | 430 | 240 | 280 |  |
| 78 | 380 | 300 | 280 | B |
| 93 | 400 | 320 | 320 |  |
|  |  |  |  |  |
|  | **Titres (PFU/ml, standardized to tube A)** | | | |
|  |  |  |  |  |
| Adsorption control | 200 | 0 | 250 |  |
| 3 | 1350 | 1300 | 1650 |  |
| 18 | 1250 | 1200 | 1500 | A |
| 33 | 1500 | 1200 | 1500 |  |
| 48 | 1400 | 1050 | 1450 |  |
| 58 | 4050 | 2000 | 1900 |  |
| 63 | 13500 | 9000 | 7000 |  |
| 68 | 21500 | 12000 | 14000 |  |
| 78 | 19000 | 15000 | 14000 | B |
| 93 | 20000 | 16000 | 16000 |  |
|  |  |  |  |  |
| **Burst size = Average B / (Average A - Adsorption control)** |  |  |  |  |
|  |  |  |  |  |
|  | R1 | R2 | R3 |  |
| **Average B** | 20166.67 | 14333.33 | 14666.66667 |  |
| **Average A - Ads control** | 1175 | 1187.5 | 1275 |  |
|  |  |  |  |  |
| **Burst size** | 17.16312 | 12.07018 | 11.50326797 |  |
|  |  |  |  |  |
| *Average A and Average B were calculated based on the boxed counts  **The values highlighted in light gray were obtained from tube A. The values highlighted in dark gray were obtained from tube B. | | |  |  |


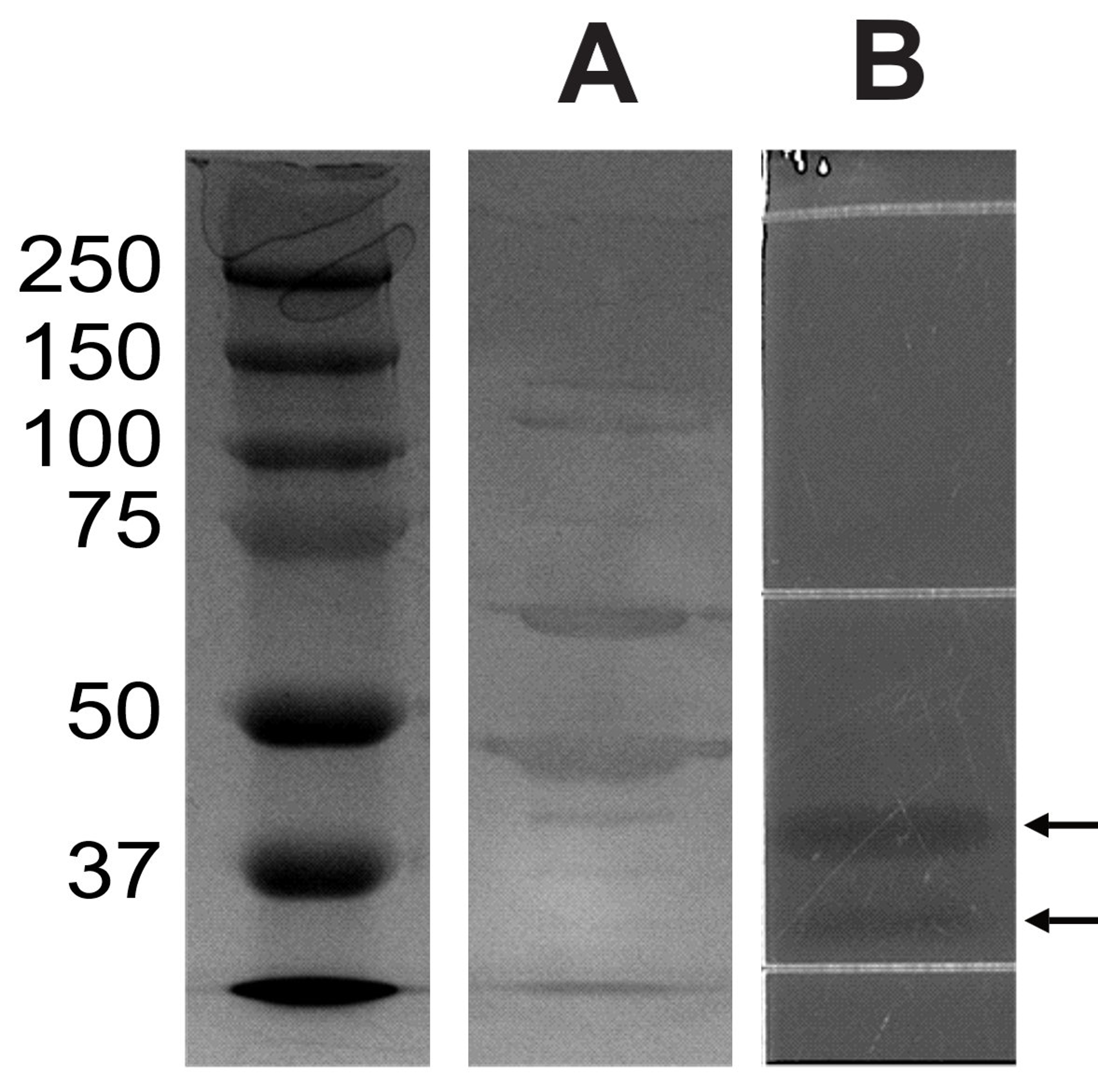


**Figure S1.** Phage lysate was purified by two rounds of CsCl centrifugation and concentrated to a titer of 2×10^12^PFU/ml. Phage proteins were separated by SDS-PAGE loading 2×10^10^PFU per lane on an 8% polyacrylamide gel. Leftmost lane: protein standard. A) Purified phage suspension loaded onto 8% SDS-PAGE gel. B) Purified phage suspension loaded onto 8% SDS-PAGE zymogram gel containing heat-killed *Lm*19111 as substrate after 20 hours renaturation. Renaturation was performed as described previously (34).

**Table S2**. Frequency of CKA15-resistant *Lm*19111.

| **Control** | **Counts at -5** | **Volume spotted (ml)** | **Concentration (CFU/ml)** |
| --- | --- | --- | --- |
| R1 | 20,29,30,22 | 0.005 | 5.05 × 10^8^ |
| R2 | 46,20 | 0.005 | 6.6 × 10^8^ |
| R3 | 30,32,34 | 0.005 | 6.4 × 10^8^ |
| R4 | 11,9 | 0.005 | 6.93 × 10^8^ |
| **Treatment** | **Counts at -1** | **Volume spotted (ml)** | **Concentration (CFU/ml)** |
| T0 | 20,22,21,21 | 0.005 | 4.2 × 10^4^ |
| T1 | 7,7 | 0.005 | 1.4 × 10^4^ |
| T3 | 17,18,18 | 0.005 | 3.53 × 10^4^ |
| T5 | 10,6,7 | 0.005 | 1.53 × 10^4^ |
|  |  |  |  |
| **Mean frequency** | 4.54 × 10^-5^ |  |  |
| **Standard deviation** | 2.97 × 10^-5^ |  |  |
|  |  |  |  |
|  |  |  |  |

*Lm*19111 were grown to mid-exponential phase in quadruplicate in cBHI, diluted to an optical density of 0.55 and 0.9 ml of bacterial suspension were mixed with either 0.1ml of PBS (control) or with 0.1ml of phage suspension at a titre of 1.17 × 10^11^PFU/ml (test) and incubated for 10 minutes at 30°C to allow time for adsorption to occur. The frequency of resistant mutants is calculated by dividing the treatment CFU/ml by the control CFU/ml of the same biological replicate. Calculation of mean frequency and standard deviation was performed in Excel.

**Table S3.** Counts of *Lm*19111 at different times after addition of phage CKA15 to exponentially growing *Lm*1911.

| Time (minutes) | Counts | Dilution | Concentration (CFU/ml) |
| --- | --- | --- | --- |
| T0 | 3,8 | -6 | 5.5× 10^8^ |
| T1 | 2,5 | -6 | 3.5× 10^8^ |
| T3 | 7,13 | -4 | 10^7^ |
| T5 | 11,9 | -4 | 10^7^ |

Interpretation:

The proportion of bacterial survivors at T3 and T5 is 0.0$\underline{18}$.

$$proportion survivors=e^{-MOI}$$

$$0.0182= e^{-MOI}$$

$$ln \left( 0.0182 \right) = -MOI$$

$$MOI=4.01$$

**
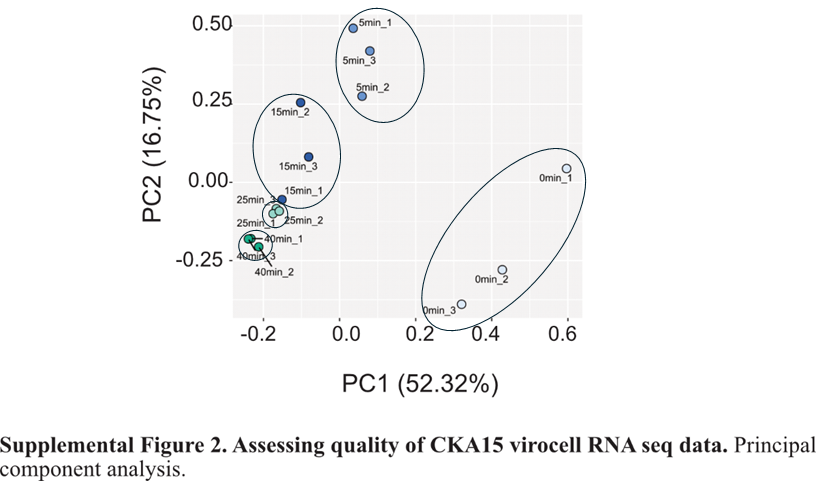
**

**Figure S2. Assessing quality of CKA15 virocell RNAseq data.** Principal component analysis was performed using FPKM-normalized count data from the Illumina-based RNAseq experiment after *In silico* removal of rRNA reads. Analysis was performed in R using ggfortify and ggrepel libraries. Ovals were added manually to show clustering of replicates from the different time points.


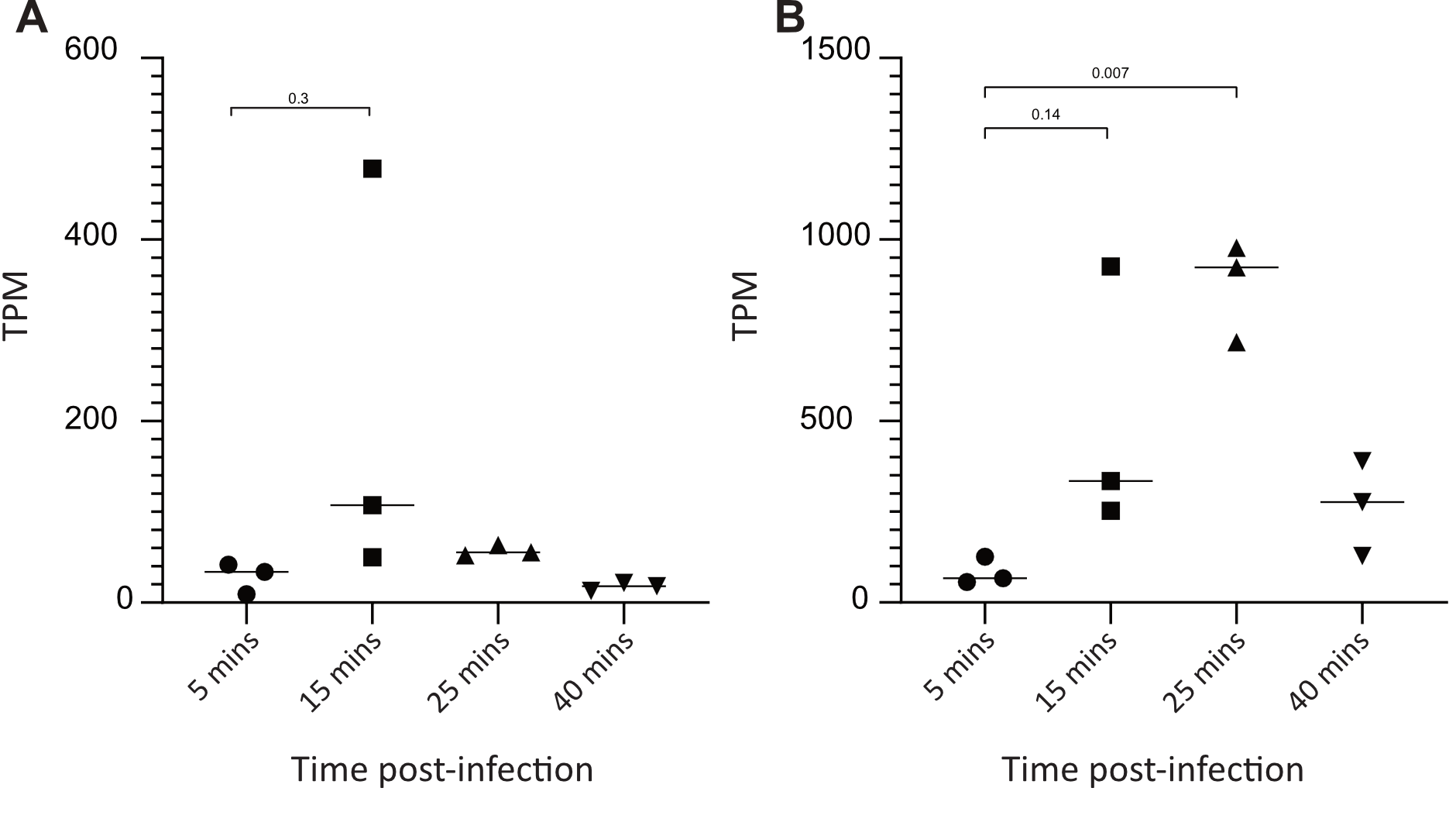


**Figure S3. Abundance of phage sigma and anti-sigma factor gene transcripts post-infection.** TPM-normalized transcript abundance of predicted anti-sigma factor (A) and sigma factor (B). Graphs were made in GraphPad Prism 8.4.3. An ordinary one-way ANOVA was performed with Turkey’s multiple comparisons test.

**Table S4.** SRA accession numbers of raw sequence data generated for this work.

| **Accession** | **Sample Name** |
| --- | --- |
| SAMN44083784  SAMN44083787  SAMN44083788  SAMN44083789  SAMN44083790  SAMN44083791  SAMN44083792  SAMN44083793  SAMN44083794  SAMN44083795  SAMN44083796  SAMN44083797  SAMN44083798  SAMN44083799  SAMN44083800 | CKA15_ont_cap_control_reads |
| SAMN44083785 | CKA15_ont_cap_enriched_reads |
| SAMN44083786 | Sample_1_T0_R1 |
| SAMN44083787 | Sample_2_T5_R1 |
| SAMN44083788 | Sample_3_T15_R1 |
| SAMN44083789 | Sample_4_T25_R1 |
| SAMN44083790 | Sample_5_T40_R1 |
| SAMN44083791 | Sample_6_T0_R2 |
| SAMN44083792 | Sample_7_T5_R2 |
| SAMN44083793 | Sample_8_T15_R2 |
| SAMN44083794 | Sample_9_T25_R2 |
| SAMN44083795 | Sample_10_T40_R2 |
| SAMN44083796 | Sample_11_T0_R3 |
| SAMN44083797 | Sample_12_T5_R3 |
| SAMN44083798 | Sample_13_T15_R3 |
| SAMN44083799 | Sample_14_T25_R3 |
| SAMN44083800 | Sample_15_T40_R3 |
